# Hepatoprotective effects of oyster-derived bioactive compounds in alcoholic liver disease: a systematic review

**DOI:** 10.3389/fgstr.2026.1737942

**Published:** 2026-03-17

**Authors:** Rui Chen, Yanan Qin, Ping Yu

**Affiliations:** 1 Clinical Nutrition Department, Yantaishan Hospital, Yantai, Shandong, China; 2 Women’s Health Department, Zhaoyuan Maternal and Child Health Hospital, Yantai, Shandong, China

**Keywords:** alcoholic liver disease, gut–liver axis, inflammation, oxidative stress, oyster-derived bioactives, peptides, polysaccharides

## Abstract

**Background:**

Alcoholic liver disease (ALD) is a major global cause of liver-related morbidity and mortality, driven by excessive alcohol consumption and characterized by oxidative stress, inflammation, disordered lipid metabolism, and gut–liver axis dysfunction. Oyster-derived bioactive compounds have shown hepatoprotective potential in experimental settings; however, their efficacy and role in ALD management remain unclear.

**Objective:**

To systematically evaluate and synthesize preclinical and clinical evidence on oyster-derived bioactive compounds for the prevention and treatment of ALD.

**Methods:**

PubMed, Web of Science, and Scopus were searched for studies examining oyster-derived bioactives, including polysaccharides, peptides, protein hydrolysates, and related extracts, in alcohol-induced liver injury models. Two reviewers independently screened studies and extracted data. Risk of bias was assessed using the SYRCLE tool for animal studies and RoB 2.0 for human trials. Certainty of evidence was evaluated using the GRADE framework.

**Results:**

Eleven studies met the inclusion criteria, comprising ten animal studies and one randomized controlled trial. In animal models, oyster-derived interventions reduced alanine and aspartate aminotransferase levels by approximately 34-56%, increased antioxidant defenses (glutathione and superoxide dismutase increased by up to 45% and 40%, respectively), and decreased inflammatory mediators including TNF-α, IL-1β, and IL-6. Improvements in lipid metabolism and gut–liver axis markers were also reported in several studies. The single human trial demonstrated a modest reduction in γ-glutamyl transferase, with no significant changes in ALT or AST. Overall, the certainty of evidence ranged from very low to low, reflecting methodological heterogeneity, risk of bias, and limited human data.

**Conclusions:**

Oyster-derived bioactives consistently demonstrate hepatoprotective effects in preclinical models of ALD through antioxidant, anti-inflammatory, metabolic, and gut-mediated mechanisms. However, the current evidence base is preliminary, and well-designed, adequately powered clinical trials are required to determine their clinical efficacy, optimal formulation, and long-term safety.

**Systematic Review Registration:**

https://www.crd.york.ac.uk/PROSPERO/view/CRD420251104584, identifier CRD420251104584.

## Introduction

Alcoholic liver disease (ALD), also referred to as alcohol-related liver disease (ARLD) in recent clinical guidelines, is a leading cause of liver-related morbidity and mortality worldwide. Excessive and sustained alcohol consumption is the principal etiological factor, and ALD accounts for a substantial proportion of cirrhosis and hepatocellular carcinoma cases globally, as well as representing one of the most common indications for liver transplantation in Europe and the United States (1).

Clinically, ALD encompasses a broad disease spectrum, ranging from hepatic steatosis (alcoholic fatty liver), which is potentially reversible with sustained abstinence, to progressive fibrosis, cirrhosis, alcoholic hepatitis, and ultimately hepatocellular carcinoma (2, 3). Although early-stage steatosis may regress, continued alcohol exposure accelerates disease progression, leading to irreversible structural and functional liver injury. Despite its high prevalence and clinical burden, the complex pathophysiology of ALD remains incompletely understood, limiting the development of effective disease-modifying therapies (4).

The onset and progression of ALD are driven by several interrelated biological mechanisms. A central feature is oxidative stress, arising from excessive production of reactive oxygen species during ethanol metabolism, which overwhelms endogenous antioxidant defenses and promotes lipid peroxidation, mitochondrial dysfunction, and hepatocyte apoptosis (5, 6). Chronic alcohol exposure also activates innate immune responses, particularly Kupffer cells, leading to sustained overproduction of pro-inflammatory cytokines such as tumor necrosis factor-α (TNF-α), interleukin (IL)-6, and IL-1β, thereby amplifying hepatic inflammation and tissue injury (6, 7). In parallel, alcohol disrupts hepatic lipid metabolism, promoting triglyceride accumulation and exacerbating steatosis (8). These processes interact to establish a self-perpetuating cycle of oxidative damage, inflammation, metabolic dysregulation, and fibrogenesis that underlies ALD progression (4).

Current therapeutic options for ALD remain limited. Clinical management relies primarily on supportive care, counseling for sustained alcohol abstinence, nutritional optimization, and corticosteroid therapy in selected cases of severe alcoholic hepatitis (9). These strategies are often associated with variable efficacy, potential adverse effects, and limited impact on the underlying pathogenic mechanisms. As a result, there is a pressing need to identify safe, accessible, and biologically targeted interventions that can complement existing approaches, particularly in early or moderate stages of disease.

In this context, natural bioactive compounds have received increasing attention due to their antioxidant, anti-inflammatory, and lipid-modulating properties. Among these, oyster-derived bioactives have emerged as promising candidates. The Pacific oyster (*Crassostrea gigas*), one of the most widely cultivated shellfish species worldwide, contains a diverse array of biologically active components, including polysaccharides, peptides, protein hydrolysates, glycogen, and taurine-rich fractions. Polysaccharides alone account for approximately 22–36% of oyster dry weight (10). Experimental studies suggest that oyster-derived preparations can attenuate ethanol-induced liver injury by reducing oxidative stress, modulating inflammatory responses, improving lipid metabolism, and enhancing endogenous antioxidant systems (11, 12). Proposed mechanisms include upregulation of enzymes such as superoxide dismutase and glutathione peroxidase, suppression of malondialdehyde accumulation, and downregulation of pro-inflammatory cytokines and signaling pathways (13).

Despite these encouraging findings, the molecular mechanisms and translational relevance of oyster-derived hepatoprotection remain insufficiently defined. Existing studies are fragmented across heterogeneous experimental models, extraction methods, and bioactive compositions, and results vary depending on whether polysaccharide-rich extracts, peptide hydrolysates, or other oyster-derived preparations are examined. To date, no systematic review has comprehensively synthesized both preclinical and clinical evidence on oyster-derived bioactives specifically in the context of alcohol-induced liver injury and ALD.

To address this knowledge gap, we conducted a systematic review of available animal and human studies investigating oyster-derived polysaccharides, peptides, protein hydrolysates, and related bioactives in ethanol-induced models of liver injury and ALD. The objectives of this review were to critically appraise methodological quality, synthesize mechanistic and therapeutic evidence across bioactive classes and disease models, and evaluate the potential role of oyster-derived compounds as preventive or adjunctive strategies for alcohol-related liver disease.

## Methods

### Protocol and reporting standards

This systematic review was conducted in accordance with the Preferred Reporting Items for Systematic Reviews and Meta-Analyses (PRISMA) 2020 guidelines. The protocol and methodology were developed following established guidance for systematic reviews of preclinical and translational research and were prospectively registered in PROSPERO (CRD420251104584).

### Eligibility criteria

The objective of this systematic review was to evaluate current evidence on the mechanisms and effects of oyster-derived bioactive compounds in the prevention and/or treatment of alcoholic hepatitis and alcohol-induced liver injury. For the purposes of this review, oyster-derived bioactives were defined broadly to include polysaccharides, protein–polysaccharide complexes, peptide-rich hydrolysates, glycogen-rich extracts, taurine-containing preparations, and standardized oyster-derived dietary supplements. This definition was applied consistently across study screening, data extraction, and evidence synthesis to ensure alignment between the review objectives and included studies.

### Inclusion and exclusion criteria

Studies were eligible if they evaluated animals or humans with ethanol- or alcohol-induced liver injury or alcoholic liver disease and examined the effects of oyster-derived bioactive interventions. Eligible comparators included placebo, vehicle, conventional hepatoprotective agents such as silymarin, or no intervention in uncontrolled studies. Outcomes of interest included hepatic injury biomarkers such as alanine aminotransferase, aspartate aminotransferase, γ-glutamyl transferase, and histological findings; oxidative stress markers including malondialdehyde, glutathione, superoxide dismutase, and catalase; inflammatory mediators and signaling pathways such as tumor necrosis factor-α, interleukin-1β, interleukin-6, and nuclear factor-κB; indices of lipid metabolism; gut–liver axis–related outcomes including microbiota composition and intestinal barrier markers; and safety outcomes such as adverse events or toxicity. Eligible study designs included *in vivo* animal experiments, randomized controlled trials, pilot trials, and observational human studies. Studies were excluded if they were solely *in vitro*, if they were editorials, letters, or narrative reviews, or if they were published in a language other than English. Reference lists of excluded reviews were screened to identify additional eligible primary studies.

### Information sources and search strategy

PubMed, Web of Science, and Scopus were systematically searched from database inception to June 30, 2025. Full search strategies for all databases are provided in **Supplementary Material S1**. The PubMed search combined terms related to alcoholic liver disease (“alcoholic liver disease,” “alcohol-induced liver injury,” or “alcoholic hepatitis”) with oyster-related terms (“oyster,” “Crassostrea,” “oyster polysaccharide,” “oyster peptide,” or “oyster hydrolysate”). Equivalent strategies were adapted for the other databases. Additional studies were identified through citation tracking and screening of relevant review articles.

### Interventions

The primary interventions of interest were oyster-derived bioactive preparations administered in the context of alcohol- or ethanol-induced liver injury. These included polysaccharide-rich extracts, protein–polysaccharide complexes, peptide-rich hydrolysates, glycogen- or taurine-rich extracts, and standardized oyster-derived dietary supplements. Where reported, detailed information was extracted regarding the route and schedule of administration, extraction method, oyster species, dose and duration of treatment, and timing of intervention relative to alcohol exposure. For controlled studies, comparators included placebo, vehicle, standard care, or positive controls such as silymarin. Uncontrolled studies without formal comparators were retained but identified as such during risk of bias assessment.

### Study selection

All retrieved records were imported into Mendeley, and duplicate entries were removed. Two reviewers independently screened titles and abstracts against the eligibility criteria, followed by full-text review of potentially relevant studies to determine final inclusion. Disagreements were resolved through discussion or, when necessary, by consultation with a third reviewer. The study selection process was documented using a PRISMA flow diagram.

### Data extraction

Two reviewers independently extracted data from each included study using a standardized extraction form. Extracted information included study characteristics; population or animal model details; alcohol exposure protocols; intervention characteristics including bioactive type, oyster species, extraction method, dose, route, timing, and duration; comparator characteristics; outcome measures; reported adverse events; and mechanistic findings related to molecular pathways or gut–liver axis effects. Outcomes were categorized as primary or secondary according to the protocol. Discrepancies were resolved through discussion or third-reviewer adjudication, and corresponding authors were contacted when key information was missing or unclear.

### Risk of bias assessment

Risk of bias was assessed independently by two reviewers. Inter-rater agreement was quantified using Cohen’s κ statistic, which indicated strong agreement (κ = 0.82). The SYRCLE Risk of Bias tool was used for animal studies, assessing sequence generation, allocation concealment, random housing, blinding, incomplete outcome data, selective reporting, and other sources of bias. The Cochrane Risk of Bias 2.0 tool was used for the single randomized controlled trial, evaluating the randomization process, deviations from intended interventions, missing outcome data, outcome measurement, and selection of reported results. No *in vitro* studies met the inclusion criteria; therefore, the *in vitro* risk of bias checklist specified in the protocol was not applied. Domain-level judgments were categorized as low, high, or unclear risk of bias, and overall study-level risk was assigned accordingly.

### Data synthesis

Because of substantial heterogeneity in study design, animal models, intervention types, dosing regimens, and outcome measures, quantitative meta-analysis was not performed. Instead, a structured narrative synthesis was conducted, organized by outcome domain, including liver injury markers, oxidative stress, inflammation, lipid metabolism, gut–liver axis outcomes, and safety. Where feasible, effect estimates were summarized as percentage changes relative to alcohol-only control groups.

### Certainty of evidence

The certainty of evidence for major outcomes was evaluated using the GRADE framework, taking into account risk of bias, inconsistency, indirectness, imprecision, and potential publication bias.

## Results

The systematic search identified eleven studies that met the inclusion criteria, comprising one human randomized controlled trial and ten *in vivo* animal studies (**Table 1**; **Supplementary Table 1**) (10, 12, 14–22). The included studies were conducted predominantly in East Asia, with seven from China, three from Korea, and one from Japan.

**Table 1:** Study characteristics and methodological details of included studies investigating oyster-derived interventions for alcohol-related liver disease.

Author, year	Country	Study type/ALD model	Model/Population	Duration	N (groups)	Control(s)
Osaki, 2015 (14)	Japan	Human RCT	Adults, habitual drinkers (GGT 50–150 IU/L)	12 wks	84 (42/group; 74 completed)	Placebo
Jiang, 2021 (15)	China	Animal (chronic ethanol feeding)	C57BL/6 mice, EtOH-induced injury	5 wks (4 wks + 28 d EtOH)	40 (4×10)	Lieber–DeCarli (−EtOH)
Shi, 2015 (16)	China	Animal (chronic ethanol gavage)	BALB/c mice, chronic EtOH model*	14 d	48 (6×8 per model)	Saline; Tiopronin
Zhang, 201 (17)	China	Animal (chronic ethanol gavage)	Wistar rats, ALD (EtOH gavage)	9 wks	75 (5×15)	Distilled water
Zhao, 2019 (18)	China	Animal (chronic ethanol gavage)	Kunming mice, EtOH-induced injury	35 d	40 (5×8)	Saline
Lee, 2021 (12)	Korea	Animal (acute ethanol + co-injury)	ICR mice, acute EtOH & D-GalN	7 d/single dose	NR (varied by arm)	Saline/water
Wang et al., 2022 (OPH) (22)	China	Animal (chronic ethanol diet)	C57BL/6J mice, chronic EtOH diet	9 wks	72 (6×12)	Lindros control diet
Wang et al., 2022 (OP) (22)	China	Animal (chronic ethanol gavage)	C57BL/6 mice, chronic EtOH model	6 wks	60 (6×10)	Saline
Siregar, 2022 (19)	Korea	Animal (acute ethanol binge)	C57BL/6 mice, single EtOH binge	7 h post-binge	Varies by assay	Vehicle
Byun, 2021 (20)	Korea	Animal (chronic ethanol diet)	Sprague–Dawley rats, EtOH diet	10 wks (6 + 4)	50 (5×10)	Iso-caloric control diet
Gao, 2022 (21)	China	Animal (chronic ethanol feeding)	C57BL/6 mice, chronic EtOH	4 wks	60 (6×10)	Normal; Model; Silymarin

*BALB/c mice received parallel chronic ethanol injury protocols as described in the original study.

The human evidence consisted of a randomized, double-blind, placebo-controlled trial conducted in Japan. This study evaluated oyster extract supplementation in 84 habitual alcohol consumers with elevated γ-glutamyl transferase (GGT; 50–150 IU/L) over a 12-week intervention period (14).

The ten animal studies included eight mouse models and two rat models. Various strains were used, including C57BL/6, Kunming, ICR, Sprague–Dawley, and Wistar. Experimental designs encompassed both acute and chronic alcohol exposure models, with intervention durations ranging from 7 hours (acute single-dose or binge models) to 12 weeks (chronic ethanol feeding or gavage protocols). Group sizes typically ranged from 8 to 15 animals, with total study populations between 40 and 75 animals (10, 12, 15–22).

All animal studies incorporated control groups, most commonly vehicle- or saline-treated controls, and several included positive comparators such as silymarin, atorvastatin, or dimethyl diphenyl bicarboxylate. Randomization procedures were reported in most studies; however, blinding of caregivers and outcome assessors was infrequently described.

Detailed methodological characteristics, including inclusion and exclusion criteria, housing and husbandry conditions, allocation procedures, and blinding status, are summarized in **Supplementary Table 1**.

### Extract characteristics

This review included eleven studies evaluating oyster-derived bioactive interventions for alcohol-related liver disease, encompassing a range of oyster species, extraction approaches, and bioactive fractions (**Table 2**; **Supplementary Table 2**). *Crassostrea gigas* was the most frequently studied species, accounting for 81.8% of included studies (9/11), while *Crassostrea talienwhanensis* and *Ostrea rivularis* were each investigated in one study (9.1% each).

**Table 2:** Overview of oyster-derived bioactive extractions and experimental protocols in included studies.

Author(s), year	Oyster species	Extraction method	Bioactive fraction (dominant)	Dose (range)	Duration	ALD induction model
Osaki, 2015 (14)	C. gigas	Powdered whole extract	Glycogen-rich fraction	1,000 mg/day	12 wks	Habitual alcohol consumption
Jiang, 2021 (15)	C. gigas	Hot-water extraction + purification	Polysaccharides (RPS, SPS; glucose-rich)	282 mg/kg	4 wks	Lieber–DeCarli diet + acute EtOH gavage
Shi, 2015 (16)	C. gigas	Hot-water extraction	Polysaccharide CGPS-1 (β-glucan)	50–450 mg/kg	14 d	Chronic EtOH gavage
Zhang, 2014 (17)	C. gigas (mixed mollusks)	Granulated oyster extract	Taurine + polysaccharides	0.12–1.20 g/kg	8 wks	Escalating intragastric EtOH
Zhao, 2019 (18)	C. gigas	Chemical sulfation of polysaccharide	Sulfated glucan	100–400 mg/kg	35 d	Intragastric EtOH
Lee, 2021 (12)	C. gigas	Raw vs subcritical water extraction	Glucose-based polysaccharides	250 mg/kg	Single dose/7 d	EtOH binge + D-GalN
Wang et al., 2022 (22)	C. gigas	Enzymatic hydrolysis	Protein hydrolysates (<2 kDa peptides)	200–800 mg/kg	8 wks	Chronic ethanol diet
Wang et al., 2022 (22)	C. talienwhanensis	Enzymatic hydrolysis	Oyster peptides (<3.5 kDa)	120–480 mg/kg	6 wks	Daily EtOH gavage
Siregar, 2022 (19)	C. gigas	Concentration of oyster broth	Taurine-rich extract	200 mg/kg	Single dose	EtOH binge
Byun, 2021 (20)	C. gigas	Enzymatic hydrolysis	TGPN hydrolysate (YA dipeptide-rich)	50–200 mg/kg	4 wks (post-EtOH)	Lieber–DeCarli diet
Gao, 2022 (21)	C. gigas	Enzymatic hydrolysis	Protein hydrolysates (<2 kDa peptides)	200–800 mg/kg	4 wks	Chronic ethanol diet

Extraction Method refers to the technical process used to obtain the preparation, while Bioactive Fraction denotes the dominant chemical constituents evaluated biologically.

To improve clarity and consistency, extraction method and bioactive composition were distinguished as separate classification domains. Extraction methods included conventional hot-water extraction, enzymatic hydrolysis, and more advanced approaches such as subcritical water extraction (175 °C, 60 bar) and ethanol-free isoelectric precipitation. These procedures yielded chemically distinct bioactive fractions rather than single purified compounds.

Across studies, polysaccharide-dominant fractions were the most commonly investigated bioactive class (54.5%, 6/11 studies), followed by protein hydrolysates or peptide-enriched fractions (36.4%, 4/11 studies). One study evaluated a glycogen-rich oyster extract (9.1%). Polysaccharide fractions were primarily characterized by monosaccharide composition (e.g., glucose, rhamnose, galacturonic acid), molecular weight distribution, and degree of sulfation, whereas peptide-based preparations were defined by molecular weight cut-offs (<2–3.5 kDa) and enrichment of specific dipeptides or short peptides.

Dosing regimens varied substantially across studies, although oral administration was consistently employed. Administered doses ranged from 50 mg/kg to 1,200 mg/kg body weight in animal models, with exposure durations spanning from single-dose or short-term interventions to chronic administration for up to 12 weeks. Most studies (63.6%, 7/11) used gavage-based administration (intragastric or oral), while three studies (27.3%) delivered interventions via dietary supplementation or tablets. One study did not specify the administration route.

Alcohol-related liver disease induction protocols predominantly involved chronic ethanol exposure (63.6%, 7/11 studies), including Lieber–DeCarli liquid diet models and progressive intragastric ethanol gavage regimens. Ethanol concentrations ranged from 5% to 50% (v/v), with exposure durations between 14 days and 8 weeks. Acute or binge-type models were used in the remaining studies, often combined with hepatotoxic co-insults such as D-galactosamine. These approaches collectively represent established experimental paradigms for modeling alcohol-induced liver injury.

Detailed information on extraction procedures, physicochemical characterization (including molecular weight, monosaccharide composition, and sulfation degree), and co-inducing factors is provided in **Supplementary Table 2**.

### Liver function enzymes and hepatoprotective efficacy

In the randomized, double-blind, placebo-controlled trial (14), supplementation with an oyster-derived extract for 12 weeks resulted in a statistically significant reduction in serum γ-glutamyl transferase (GGT) compared with placebo at week 12 (p = 0.049). Mean GGT levels decreased by approximately 8% in the intervention group, whereas an increase of approximately 12% was observed in the placebo group. In contrast, alanine aminotransferase (ALT) and aspartate aminotransferase (AST) showed small numerical reductions in the intervention group and slight increases in the placebo group, but these differences did not reach statistical significance.

Across preclinical studies (10, 12, 15–22), oyster-derived interventions consistently attenuated ethanol-induced elevations in hepatic injury enzymes. Reductions in ALT and AST were observed following administration of raw and steamed oyster polysaccharides (15), with enzyme improvements correlating with increased abundance of *Lactobacillus reuteri*. The CGPS-1 polysaccharide fraction produced dose-dependent decreases in ALT and AST (16), while oyster extract granules significantly reduced transaminase levels across low-, medium-, and high-dose groups in chronic ethanol models (17). Sulfated polysaccharides were associated with pronounced reductions in ALT and AST at higher doses (18), and both raw and subcritical water-extracted oyster powders lowered enzyme levels in acute and chronic injury models (12).

Peptide-based preparations demonstrated some of the largest quantitative effects. Oyster protein hydrolysates reduced ALT by 34.1% and AST by 35.3%, accompanied by a 17.2% reduction in alkaline phosphatase (ALP) and a 17.3% increase in total protein (22). Oyster peptide fractions decreased AST by 56.3%, ALT by 47.0%, and GGT by 46.3% relative to ethanol controls (10). Administration of taurine-rich oyster broth concentrate also resulted in significant reductions in ALT and AST (19). TGPN hydrolysates restored ALT and AST levels toward normal in a dose-dependent manner (20), while oyster protein hydrolysates in chronic ethanol models reduced ALT, AST, and lactate dehydrogenase (LDH) and enhanced alcohol dehydrogenase (ADH) activity (21). Collectively, these findings indicate consistent hepatoprotective effects across diverse oyster-derived bioactive preparations (**Table 3**; **Supplementary Table 3**).

**Table 3:** Effects of oyster-derived bioactives on liver injury markers in alcohol-related liver disease models.

Author(s), year	ALT	AST	Other enzymes	Key outcome
Osaki, 2015 (human) (14)	−10% (not significant)	−10% (not significant)	GGT −8% vs +12% placebo (p = 0.049)	Only human trial; modest biochemical effect
Jiang, 2021 (15)	Significant reduction (p < 0.05)	Significant reduction (p < 0.05)	Not reported	Polysaccharides (RPS, SPS) improved transaminases
Shi, 2015 (16)	Significant reduction (p < 0.05–0.01)	Significant reduction (p < 0.05)	Not reported	Dose-dependent hepatoprotection
Zhang, 2014 (17)	Significant reduction (p < 0.05–0.01)	Significant reduction (p < 0.05–0.01)	Not reported	Dose-dependent transaminase lowering
Zhao, 2019 (18)	Significant reduction (p < 0.01)	Significant reduction (p < 0.01)	Not reported	Strong effect at higher doses
Lee, 2021 (12)	Reduction observed (not statistically tested)	Reduction observed (not statistically tested)	Not reported	Enzyme trends supported protection
Wang et al., 2022 (OPH) (22)	−34.1% (p < 0.01)	−35.3% (p < 0.01)	ALP −17.2% (p < 0.05); TP + 17.3% (p < 0.05)	Significant liver protection
Wang et al., 2022 (OP) (22)	−47.0% (p < 0.01)	−56.3% (p < 0.01)	GGT −46.3% (p < 0.01)	Largest enzyme reductions
Siregar, 2022 (19)	Significant reduction (p < 0.05)	Significant reduction (p < 0.05)	Not reported	Taurine-rich broth protective
Byun, 2021 (20)	Dose-dependent normalization (p < 0.001)	Dose-dependent normalization (p < 0.001)	Not reported	Enzymes returned toward control levels
Gao, 2022 (21)	Significant reduction (p < 0.01)	Significant reduction (p < 0.01)	LDH reduced; ADH increased (p < 0.05)	Comparable efficacy to silymarin

Percentage changes are reported where available. When exact values were not provided, statistically significant directional changes relative to ethanol controls are described. “Not significant” indicates p ≥ 0.05.

### Lipid profile and oxidative stress indicators

**Figure 1:**
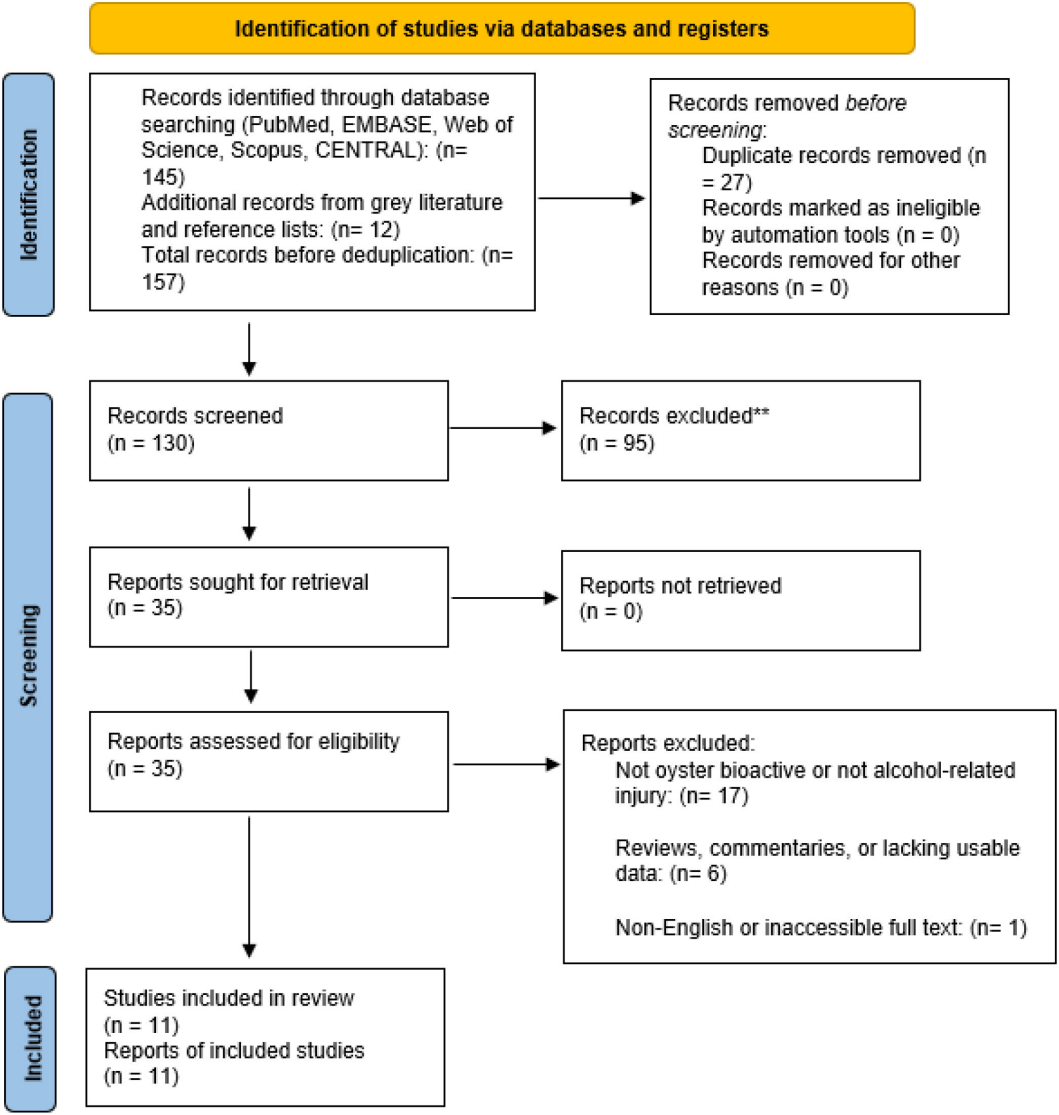
PRISMA flow diagram.

Across the included studies, oyster-derived interventions were associated with improvements in lipid metabolism and reductions in oxidative stress markers (**Table 4A**; **Supplementary Table 3**; **Figure 1**). In the single human randomized controlled trial, no statistically significant changes were observed in serum triglycerides (TG), total cholesterol (TC), high-density lipoprotein cholesterol (HDL-C), or low-density lipoprotein cholesterol (LDL-C) following oyster extract supplementation (14).

**Table 4A:** Effects of oyster-derived interventions on lipid profile and oxidative stress markers in ALD models.

Author, year	Lipid outcomes	Oxidative stress outcomes
Osaki, 2015 (14)	No significant changes in TG, TC, HDL-C, or LDL-C	Not reported
Jiang, 2021 (15)	Hepatic TG −17.9% (RPS), −15.7% (SPS); serum TG reduced	GSH, SOD, and CAT restored; MDA reduced
Shi, 2015 (16)	Not reported	MDA reduced; SOD increased (dose-dependent)
Zhang, 2014 (17)	TG and TC significantly reduced (p < 0.05–0.01)	GSH increased; MDA reduced (dose-dependent)
Zhao, 2019 (18)	TC and LDL-C significantly reduced	*In vitro* radical scavenging increased; *in vivo* oxidative markers not measured
Lee, 2021 (12)	Not reported	GSH increased; MDA reduced; antioxidant enzymes increased
Wang et al., 2022 (OP) (22)	TC −21.6%; TG −25.6%; LDL-C −27.9%; HDL-C +33.6%	Not reported
Wang et al., 2022 (OP) (22)	TG −27.4% vs ethanol model	ROS −36.4%; SOD + 40.1%; GSH + 44.9%; MDA −53.5%
Siregar, 2022 (19)	Not reported	ROS/RNS reduced; CYP2E1 reduced; catalase increased
Byun, 2021 (20)	Serum and hepatic TG and TC reduced (p < 0.05–0.001); HDL/TC ratio increased	Not reported
Gao, 2022 (21)	TC reduced in a dose-dependent manner	MDA reduced; GSH-PX and total antioxidant capacity increased

Percentage changes are reported where available. When exact values were not provided, statistically significant directional changes relative to ethanol controls are described. Absence of data indicates that the outcome was not measured or not reported.

In animal models, polysaccharide-based interventions consistently reduced hepatic and serum triglyceride concentrations, often in a dose-dependent manner. Reductions in hepatic TG of 17.9% and 15.7% were reported for rhamnose- and sulfate-containing polysaccharide fractions, respectively, alongside decreases in circulating TG levels (15). Sulfated polysaccharides and peptide-based preparations were associated with significant reductions in total cholesterol and LDL-C (18, 22), while oyster peptide fractions improved both hepatic and circulating lipid profiles in chronic ethanol exposure models (10). TGPN hydrolysates similarly reduced serum and hepatic TG and TC concentrations and improved HDL/TC ratios (20).

Oxidative stress outcomes showed consistent improvement across preclinical studies. Multiple oyster-derived extracts restored endogenous antioxidant defenses, including superoxide dismutase (SOD), catalase (CAT), and glutathione (GSH), while reducing malondialdehyde (MDA) accumulation (12, 15, 16). Oyster peptide fractions produced some of the largest reported effects, reducing reactive oxygen species (ROS) by approximately 36%, increasing SOD activity by 40%, elevating GSH levels by 45%, and decreasing MDA concentrations by more than 50% relative to ethanol controls (10). Additional studies demonstrated reductions in ROS/reactive nitrogen species (RNS), suppression of CYP2E1 expression, and enhancement of total antioxidant capacity (19, 21). Collectively, these findings indicate that oyster-derived bioactives exert consistent antioxidant effects in ethanol-induced liver injury models.

### Inflammatory and histological outcomes

Inflammatory cytokines were consistently elevated in ethanol-exposed models and were reducedfollowing oyster-derived interventions (**Table 4B**; **Supplementary Table 3**). Across studies, decreases were reported for key pro-inflammatory mediators, including TNF-α, IL-1β, and IL-6. Several studies also reported reductions in circulating LPS levels and/or suppression of NF-κB signaling, suggesting attenuation of endotoxin-driven inflammatory activation in specific models (17, 19, 22). Histological findings aligned with these biomarker changes: compared with ethanol-only controls, oyster-treated groups showed improved hepatocyte architecture, reduced steatosis and lipid droplet accumulation, and decreased necrosis and inflammatory cell infiltration on H&E and Oil Red O staining (12, 17, 21, 22). These protective effects were observed across both polysaccharide-rich and peptide/hydrolysate preparations.

**Table 4B:** Effects of oyster-derived interventions on inflammatory markers and histological outcomes in ALD models.

Author, year	Inflammatory outcomes	Histological outcomes
Osaki 2015 (14)	Not reported	Not reported
Jiang 2021 (15)	Hepatic TNF-α and IL-1β decreased; plasma LPS, TNF-α, and IL-1β decreased (vs ethanol control)	Oil Red O: lipid droplet accumulation decreased
Shi 2015 (16)	Not reported	H&E: hepatocellular necrosis and steatosis decreased
Zhang 2014 (17)	TNF-α and IL-17 decreased; complement factors C3a and C5a decreased	H&E: hepatocyte architecture improved; steatosis decreased
Zhao 2019 (18)	Not reported	H&E: inflammatory infiltration and tissue injury decreased
Lee 2021 (12)	Not reported	H&E: necrosis and vacuolization decreased
Wang et al., 2022 (OP) (22)	IL-1β, TNF-α, and TGF-β decreased; LPS decreased	H&E: liver injury grade decreased; Oil Red O area decreased (55%)
Wang et al., 2022 (OP) (22)	IL-1β decreased (50%); IL-6 decreased (30%); TNF-α decreased (47%)	H&E: lobular inflammation and steatosis decreased
Siregar 2022 (19)	TNF-α, IL-1β, and IL-6 decreased; NF-κB activation decreased; CD68-positive infiltration decreased	H&E: hepatocellular damage attenuated; Oil Red O: lipid accumulation decreased
Byun 2021 (20)	Serum and hepatic TNF-α decreased (reported p < 0.05–0.001)	H&E: lipid droplets decreased; Oil Red O: improvement toward normal
Gao 2022 (21)	Not reported	H&E and Oil Red O: hepatocyte swelling, fat droplet accumulation, and inflammation decreased

Where exact numerical effect sizes were reported by the original study, they are presented as percent change. When studies reported only statistical significance without extractable numeric values, results are summarized as “decreased vs ethanol control” with the reported p-value range (if available). “Not reported” indicates the outcome was not measured or not presented.

### Secondary therapeutic outcomes

In addition to primary biochemical and histological endpoints, several studies reportedsupplementary mechanistic and safety-related outcomes (**Table 5**; **Supplementary Table 4**). The human randomized controlled trial reported favorable tolerability, with fewer adverse events and high compliance in the oyster extract group, while noting no clinically meaningful changes in routine clinical parameters over 12 weeks (14).

**Table 5:** Secondary outcomes from included studies.

Author(s), year	Secondary outcomes	Key findings
Osaki et al., 2015 (14)	Safety and compliance	Lower adverse event frequency in the oyster extract group (7% vs 29% placebo); no significant changes in BMI, blood pressure, or routine laboratory tests; compliance >99%.
Jiang et al., 2021 (15)	Gut microbiota and metabolites	Increased relative abundance of Lactobacillus, Bifidobacterium, and Roseburia; increased fecal/serum short-chain fatty acids; increased tight junction proteins (Occludin, ZO-1); AMPK activation increased and SREBP-1c signaling reduced.
Shi et al., 2015 (16)	Structural characterization	CGPS-1 polysaccharide characterized as a β-glucan; dose-dependent improvements in liver injury and oxidative stress markers were reported.
Zhang et al., 2014 (17)	Immune modulation and nutrition	Increased CD3+ and CD4+ T-cells; reduced CD8+ T-cells; increased NK cell activity; zinc levels restored; anti-MAA-HSA IgG reduced; histological improvements reported.
Zhao et al., 2019 (18)	Metabolomics and safety	Metabolomics identified 21 biomarkers linked to amino acid and oxidative stress-related pathways; reductions reported for TBIL, LDL-C, and total cholesterol; antioxidant capacity demonstrated *in vitro*; high-dose acute toxicity not observed (LD_50_ reported >10 g/kg).
Lee et al., 2021 (12)	Alcohol metabolism and antioxidant profiling	ADH/ALDH activity increased; blood ethanol levels reduced at several time points (30–300 min); phenolic acids and amino acids identified; subcritical-water processed preparation reported as more active than raw preparation.
Wang et al., 2022 (OPH) (22)	Omics and liver function	Reduced liver index and histologic injury grade; transcriptomic/proteomic analyses identified genes and proteins related to lipid metabolism and inflammation.
Wang et al., 2022 (OP) (22)	Molecular pathways	Dose-dependent reductions in liver enzymes reported; activation of Nrf2–HO-1 antioxidant signaling; reductions in ROS with increases in antioxidant defenses (SOD and GSH) and reductions in MDA reported (numeric values provided in the original study).
Siregar et al., 2022 (19)	ER stress, apoptosis, and alcohol metabolism	Reduced ER stress-related proteins (GRP78, PERK, CHOP) and apoptosis markers (Bax/Bcl-2, caspase-3); ADH/ALDH activity increased; functional outcomes improved in the reported model.
Byun et al., 2021 (20)	Lipid metabolism regulation	AMPK/PPAR-α signaling increased; downstream lipogenic markers reduced (ACC, SREBP-1c/2, FAS, SCD1) and fatty acid oxidation markers increased (CPT-1); adiponectin increased; dose-dependent improvements in lipids and histology reported.
Gao et al., 2022 (21)	Transcriptomics and proteomics	Improvements in liver enzymes reported alongside increased ADH activity; omics analyses implicated pathways related to oxidative phosphorylation, glutathione metabolism, and PPAR signaling; histological improvements reported.

When exact numeric effect sizes were reported in the original study, they are summarized in the table. When studies reported directional or statistically significant changes without extractable numeric values, findings are described qualitatively using “increased/decreased” relative to ethanol controls (and p-values are stated if provided).

Across animal studies, additional findings clustered into several mechanistic domains. First, multiple polysaccharide-rich preparations were associated with changes in gut–liver axis measures, including shifts in microbiota composition, increased short-chain fatty acid levels, and improved intestinal barrier-related markers in chronic ethanol models. Second, metabolomics- and omics-based studies suggested modulation of pathways related to oxidative stress defense, lipid metabolism, and inflammatory signaling. Third, several interventions were linked to changes in alcohol metabolism and cellular stress responses, including altered ADH/ALDH activity and reductions in endoplasmic reticulum stress- and apoptosis-related markers in specific models (12, 19). Finally, dose–response patterns were reported in multiple experiments, with larger doses generally associated with greater improvements in the reported outcomes (10, 16–18, 20–22).

### Risk of bias

**Figure 2:**
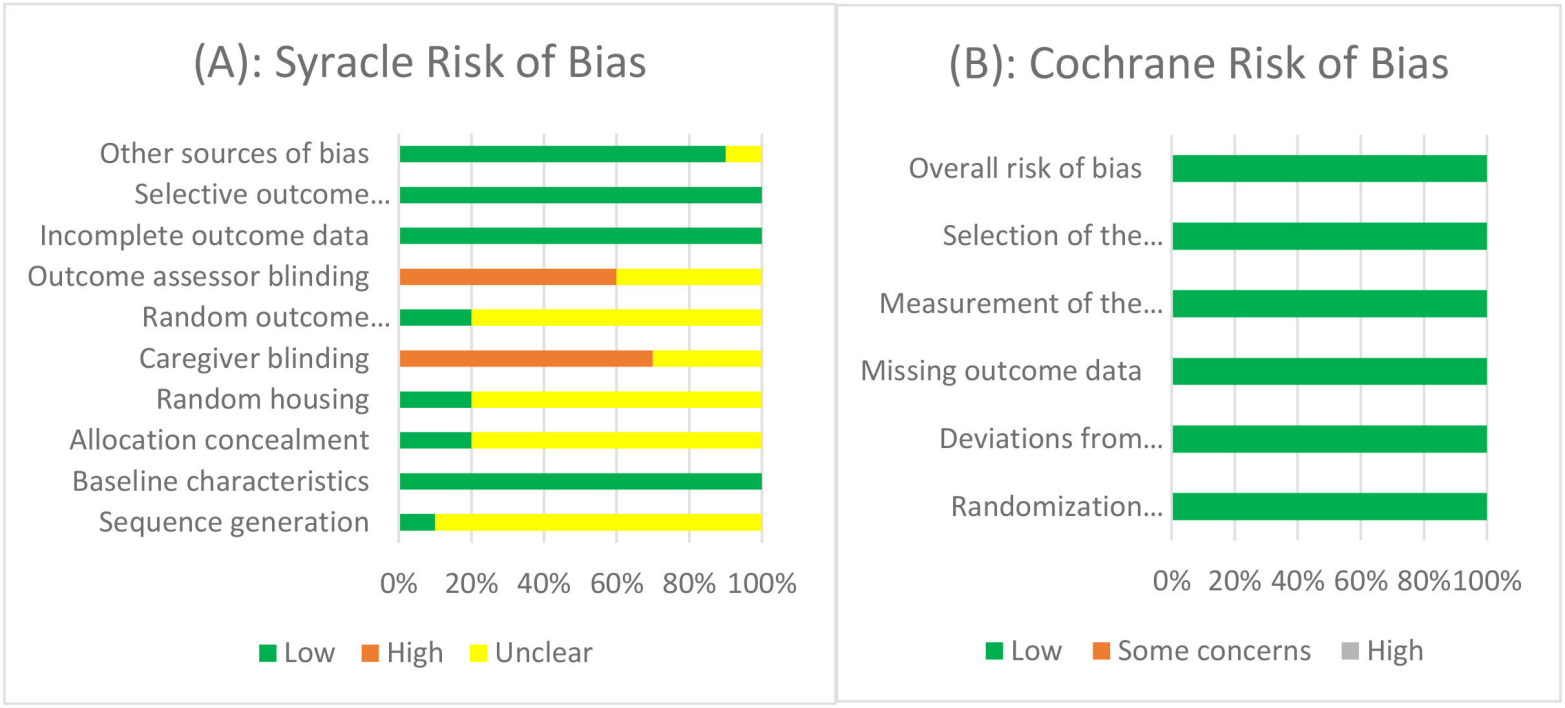
Risk of bias assessments. **(A)** SYRCLE tool results for animal studies (n = 10), showing proportions rated as low (green), unclear (yellow), or high risk (red) across bias domains. **(B)** Cochrane RoB 2.0 results for the single randomized controlled trial, rated as low risk across all domains.

Risk of bias assessments are summarized in **Figure 2** and detailed in **Supplementary Table 5**. Among the 10 animal studies evaluated with the SYRCLE tool, sequence generation and allocation concealment were seldom reported, leading to predominantly unclear judgments. Random housing was similarly underreported. High risk of bias was most frequently observed in blinding of caregivers (6 of 10 studies) and blinding of outcome assessors (4 of 10 studies). In contrast, outcome data were consistently reported across all studies, with low risk for incomplete outcome data (10/10) and selective outcome reporting (10/10). Overall, 4 studies were judged at high risk of bias, 5 at unclear risk, and only 1 at low risk. The single RCT was assessed separately using Cochrane RoB 2.0. It was rated low risk of bias across all domains, including randomization, intervention fidelity, outcome measurement, and reporting.

### Certainty of evidence (GRADE)

The certainty of evidence was evaluated using the GRADE framework (**Supplementary Table 6**). Among animal studies, evidence for liver injury biomarkers (ALT/AST and histology) was graded as Low certainty. This reflects consistent findings across all ten studies, including histological support in six, though concerns remained regarding risk of bias, indirectness, and small sample sizes. In contrast, outcomes related to oxidative stress, inflammatory markers, and gut–liver axis effects were all downgraded to Very Low certainty due to methodological limitations, variability in effect sizes, and very serious imprecision.

For human evidence, the single randomized controlled trial was also rated as Low certainty. Although well-conducted and at low risk of bias, the trial was underpowered, unreplicated, and indirect (habitual drinkers with elevated GGT rather than diagnosed ALD), limiting confidence in its clinical relevance.

Overall, while oyster-derived bioactives consistently demonstrated hepatoprotective effects in preclinical models and showed modest improvements in one clinical trial, the strength of evidence remains limited, with only two domains (ALT/AST biomarkers in animals and the single RCT) reaching Low certainty. Further adequately powered and clinically relevant trials are required before any firm conclusions can be drawn.

A statistical assessment of publication bias could not be conducted due to the insufficient number of studies per outcome (<10). However, the predominance of small, positive studies suggests that selective reporting cannot be excluded.

## Discussion

This study synthesizes results from one human randomized controlled trial and ten preclinical studies investigating oyster-derived bioactive compounds in the prevention and management of alcohol-related liver damage (ALD). Across diverse extraction methods, bioactive compositions, and animal models, the included studies reported hepatoprotective signals, including improvements in liver enzymes, lipid metabolism, antioxidant defenses, inflammatory markers, and gut-related outcomes. However, the certainty of the evidence remains low overall, and clinical applicability is uncertain given the limited human data and heterogeneity in the preclinical literature.

The most consistent finding across studies was a reduction in aminotransferases. In animal models, decreases in ALT and AST ranged from approximately 10% to 56%, suggesting reduced hepatocellular injury in ethanol-exposed animals. In the single human randomized controlled trial, oyster extract supplementation was associated with an 8% reduction in GGT, contrasted with a 12% increase in the placebo group, while ALT and AST changes were not statistically significant. Although GGT is recognized as a supportive biomarker in the context of alcohol-related liver injury and ALD progression (23, 24), the clinical significance of this relative shift is difficult to judge in the absence of defined minimal clinically important differences and in a population not diagnosed with ALD. In contrast, conventional pharmacologic treatment such as corticosteroids in severe alcoholic hepatitis can reduce short-term mortality in selected patients but has limited long-term benefit (25, 26). Therefore, the therapeutic relevance of a modest change in GGT remains uncertain, particularly for early-stage disease.

Preclinical investigations consistently reported improvements in lipid profiles. Hepatic triglyceride accumulation decreased by 15% to 48%, with oyster peptides and hydrolysates often showing larger effects within their respective models. In one study, serum total cholesterol decreased by 21.6%, LDL-C decreased by 27.9%, and HDL-C increased by 33.6%. These findings are comparable to those observed with other marine bioactives, including fucoidan and alginate oligosaccharides, which have been reported to influence lipid metabolism through AMPK activation and SREBP inhibition (27, 28). In contrast to pharmaceutical AMPK activators or fibrates, which can produce 40% to 60% reductions in triglycerides in humans (29), oyster-derived effects have not been verified in clinically defined ALD. Because steatosis is reversible in early disease, oyster bioactives may be more relevant for prevention or early-stage modulation than for advanced fibrosis or cirrhosis.

Both polysaccharides and peptides were reported to restore antioxidant defenses, including GSH, SOD, and catalase, while reducing MDA and ROS in multiple animal studies. Oyster peptides reduced ROS by 36.4% and MDA by 53.5% in the largest reported effects, consistent with reduced lipid peroxidation in ethanol models. Nonetheless, improvements in antioxidant markers do not necessarily translate into sustained clinical benefit. For example, adding N-acetylcysteine to prednisolone in alcoholic hepatitis improved 1-month survival but did not improve 6-month outcomes (30). This disconnect underscores the need for clinical trials that assess whether biomarker improvements from oyster-derived preparations translate to durable clinical and histologic outcomes.

Oyster-derived preparations were also reported to reduce inflammatory markers, including TNF-α, IL-6, and IL-1β, and some studies reported inhibition of NF-κB signaling and reduced macrophage infiltration. These findings are consistent with the broader literature on marine polysaccharides, including fucoidan, which has been reported to reduce inflammatory cytokines via MAPK and NF-κB pathway modulation (27, 28). Anti-inflammatory activity is mechanistically relevant in ALD because persistent inflammation contributes to progression from steatosis to fibrosis. However, the absence of human immunologic endpoints remains a major translational gap.

Three studies reported gut-liver axis findings, including increased Lactobacillus and Bifidobacterium, increased short-chain fatty acid production, and improved barrier integrity through increased tight junction proteins such as occludin and ZO-1. This gut-mediated pathway may be as important as direct hepatic effects because dysbiosis is a recognized feature of ALD (31, 32). A mouse study of alginate oligosaccharides reported improved intestinal barrier function with increased expression of tight junction proteins such as occludin and claudin, alongside improved microbiota composition and short-chain fatty acid production, suggesting reduced permeability and improved endotoxin handling (33). Considering active research on probiotics and fecal microbiota transplantation in ALD (31, 34, 35), oyster bioactives may represent a dietary approach for microbiome modulation. However, current evidence is limited to a small number of heterogeneous animal studies, and no human microbiome studies were identified.

### Structured interpretation by bioactive class and disease model

Although a formal subgroup analysis was not feasible, the narrative evidence suggests patterns by bioactive class and by model type. Peptide-rich hydrolysates and low-molecular-weight peptide fractions more often reported larger changes in oxidative stress markers and inflammatory signaling within their models, which is consistent with greater systemic bioavailability and direct hepatic effects. In contrast, polysaccharide-rich preparations more frequently reported gut-related endpoints, including microbiota shifts, increased short-chain fatty acids, and improved barrier markers, suggesting a stronger gut-mediated component. Model type may also influence which outcomes are detectable. Acute exposure models primarily captured short-term enzyme and oxidative stress changes, whereas chronic ethanol diet or multi-week gavage models more often evaluated steatosis, inflammatory remodeling, and gut-liver axis disruption. These patterns should be interpreted cautiously because study methods, dosing, and outcome reporting were heterogeneous across experiments.

The hepatoprotective effects likely reflect combined antioxidant, anti-inflammatory, metabolic, and gut-mediated pathways. However, the reason that different extraction methods yield broadly similar protective signals requires clearer explanation. Several non-exclusive mechanisms may account for this convergence. First, multiple preparations may share overlapping bioactive constituents that remain stable across processing methods, including taurine and certain amino acids, which may contribute to cytoprotection and oxidative stress mitigation (36). Second, different compound classes may operate through different proximal mechanisms but converge on common downstream pathways, including AMPK signaling, oxidative stress response programs, and inflammatory transcriptional regulation. Third, many preparations are multicomponent mixtures with limited characterization, and observed effects may reflect combined actions rather than a single isolated compound. Bioavailability is also a key issue. Low-molecular-weight peptides under 3.5 kDa are more likely to be absorbed systemically, whereas polysaccharides may act primarily through microbial fermentation and gut barrier effects rather than direct absorption. Pharmacokinetic studies and exposure-response experiments are needed to determine whether effective tissue concentrations can be achieved through supplementation, to clarify mechanism by compound class, and to support rational dose selection.

The hepatoprotective effects observed in this review should also be interpreted in the context of substantial biological and compositional variability among oyster-derived preparations. Most studies used extracts from Crassostrea gigas (12, 14–22), while one study used Crassostrea talienwhanensis (10). These species can differ in polysaccharide and peptide profiles, taurine content, and glycogen abundance, which may influence antioxidant capacity, NF-κB signaling, and gut-liver axis effects. Extraction methods further contributed to heterogeneity. Conventional polysaccharide preparations (15, 16, 18) and glycogen-rich powder (14) are mechanistically distinct from taurine-rich oyster broth (19), subcritical water processed extracts (12), and low-molecular-weight protein hydrolysates or peptide fractions that include <2 to 3.5 kDa peptides or YA dipeptide rich profiles (10, 20–22). Peptide-rich fractions may be more bioavailable and exert stronger systemic antioxidant and anti-inflammatory effects, while higher-molecular-weight polysaccharides may act more through gut-mediated pathways. Because only a subset of studies reported detailed chemical characterization beyond general extract labels, it remains difficult to link efficacy to specific components versus broader matrix effects. This variability limits direct comparability of effect sizes and highlights the need for standardized extraction protocols and robust characterization in future work.

Substantial methodological heterogeneity was also present across the ten animal studies and the single randomized controlled trial included in this review (10, 12, 14–22). Preclinical investigations used different species and strains, different alcohol exposure paradigms, and intervention durations ranging from a single acute exposure to multi-week regimens. Doses and dosing schedules varied widely, and outcome reporting was inconsistent across studies. This diversity precluded meaningful quantitative synthesis and limited subgroup exploration by disease stage, alcohol regimen, or extract class. As a result, we relied on structured narrative synthesis, and the directionally consistent signals should be interpreted cautiously.

Interpretation is further limited by design and reporting issues. In animal studies, factors that influence liver outcomes, such as background diet composition, pair-feeding, housing and stress, baseline metabolic status, and sex, were incompletely reported or not systematically controlled (10, 12, 15–22). Only a subset of studies clearly described randomization or blinding, and few reported stratified analyses by sex or baseline injury. In the single randomized trial, randomization and double-blinding reduce internal bias (14), but residual confounding by alcohol intake patterns, changes in drinking during follow-up, dietary patterns, concomitant medications, and metabolic comorbidities cannot be excluded. Ideally, sensitivity analyses such as excluding studies at high risk of bias, stratifying by alcohol model, or restricting comparisons to specific extract types would help clarify robustness. However, the small number of studies per outcome and incomplete reporting precluded meaningful subgroup or sensitivity analyses. These limitations are reflected in the GRADE ratings, where most outcomes were downgraded for risk of bias, inconsistency, indirectness, and imprecision.

### Clinical significance and application

The primary translational question is how oyster-derived bioactives could realistically be incorporated into ALD management. Current guidelines emphasize alcohol abstinence, nutritional support, and psychosocial interventions, with corticosteroids reserved for selected severe alcoholic hepatitis cases and liver transplantation for carefully selected patients (23–25). Oyster-derived preparations are unlikely to replace established therapies. A more plausible role is as adjunctive, food-based interventions in earlier disease stages such as steatosis or mild steatohepatitis, where reversibility is greater and the threshold for pharmacologic therapy is less favorable.

Translating preclinical evidence will require adequately powered Phase II and III trials using chemically characterized and standardized preparations, with careful attention to dose, duration, and co-interventions. Trials should enroll clearly phenotyped patients, ideally stratified by fibrosis stage and ongoing alcohol use. They should include clinically meaningful endpoints, such as changes in aminotransferases, validated noninvasive fibrosis markers, liver stiffness, and patient-reported outcomes, with prospective safety monitoring, including interactions with alcohol and medications. Given gut-liver signals in several studies (15, 18, 19), integration of microbiome and metabolomic endpoints would also be informative.

Several practical challenges should be anticipated in trial design and translation. Standardization is essential, because variability in species, extraction method, and molecular composition can change bioactivity. Dose selection is also a major challenge, because bioavailability likely differs by preparation class, and exposure-response relationships are largely unknown. In addition, confounding by changes in alcohol intake, diet, and comorbid metabolic disease can obscure true treatment effects in clinical settings. These factors argue for careful trial protocols with objective alcohol consumption measures, predefined co-interventions, and rigorous product characterization.

### Limitations of the study

This review is limited mainly by the scarcity of human clinical evidence. Only one small randomized controlled trial was available, and the remaining evidence was preclinical, limiting confidence in translation. Methodological diversity was substantial across animal studies, including extraction procedures, bioactive composition, animal strains, disease induction methods, dosing regimens, and outcome reporting. This heterogeneity prevented pooled estimates and limited inference about optimal preparations.

Risk of bias was frequently unclear or high for key SYRCLE domains, particularly sequence generation, allocation concealment, and blinding. Most outcomes were graded as very low certainty, except liver injury biomarkers in animals and the single randomized controlled trial, which were graded as low certainty. Human safety data remain limited. The single trial reported good tolerability, but long-term safety, drug-alcohol interactions, and safety in comorbid populations remain uncertain. Publication bias could not be assessed statistically due to few studies per outcome, and selective reporting cannot be excluded.

Finally, while similar protective signals were reported across polysaccharides, peptides, and hydrolysates, it is not appropriate to assume uniform mechanisms across preparations and species. This review provides the first comprehensive synthesis of oyster-derived bioactives in ALD and highlights consistent signals in antioxidant, anti-inflammatory, metabolic, and gut-liver pathways that warrant further investigation.

## Conclusion

This systematic review suggests that oyster-derived bioactives attenuate ethanol-induced liver injury in animal models via multiple mechanisms, including improved liver enzymes, lipid metabolism modulation, enhanced antioxidant defenses, reduced inflammatory signaling, and gut barrier and microbiota-related effects. However, the evidence base is limited by heterogeneity, potential bias, and sparse human data. The single human trial reported a modest improvement in GGT without significant changes in ALT or AST, emphasizing the translational gap.

Dietary equivalence statements should be avoided or framed with strong caution. Converting experimental doses into a simple amount of oyster meat does not account for extract concentration, composition, and bioavailability, and may mislead readers. Future work should prioritize dose-ranging and pharmacokinetic studies to support rational formulation and dosing. Well-powered, multi-center randomized trials with clinically meaningful endpoints and prospective safety monitoring are needed before oyster-derived bioactives can be considered for routine clinical use.

## References

[1] Danpanichkul P Duangsonk K Tham EKJ Tothanarungroj P Auttapracha T Prasitsumrit V . Increased mortality from alcohol use disorder, alcohol-associated liver disease, and liver cancer from alcohol among older adults in the United States: 2000 to 2021. Alcohol Clin Exp Res. (2025) 49:368–78. doi: 10.1111/acer.15516, PMID: 39701596 PMC11828968

[2] Liu SY Tsai IT Hsu YC . Alcohol-related liver disease: basic mechanisms and clinical perspectives. Int J Mol Sci. (2021) 22:5170. doi: 10.3390/ijms22105170, PMID: 34068269 PMC8153142

[3] Ha NB Yao F . Alcohol and hepatocellular carcinoma. Clin Liver Dis. (2024) 28:633–46. doi: 10.1016/j.cld.2024.06.007, PMID: 39362712 PMC12037205

[4] Jackson W Nguyen M . Alcohol-related liver disease. MSD Manual Professional Version. Reviewed May 2025 (2025). Available online at: https://www.msdmanuals.com/professional (Accessed January 15, 2026).

[5] Torres S Hardesty J Barrios M Garcia-Ruiz C Fernandez-Checa JC Singal AK . Mitochondria and alcohol-associated liver disease: pathogenic role and target for therapy. Semin Liver Dis. (2025) 45:180–94. doi: 10.1055/a-2421-5658, PMID: 39317216 PMC12981299

[6] Tan HK Yates E Lilly K Dhanda AD . Oxidative stress in alcohol-related liver disease. World J Hepatol. (2020) 12:332–49. doi: 10.4254/wjh.v12.i7.332, PMID: 32821333 PMC7407918

[7] Wu X Fan X Miyata T Kim A Cajigas-Du Ross CK Ray S . Recent advances in understanding of pathogenesis of alcohol-associated liver disease. Annu Rev Pathol. (2023) 18:411–38. doi: 10.1146/annurev-pathmechdis-031521-030435, PMID: 36270295 PMC10060166

[8] You M Arteel GE . Effect of ethanol on lipid metabolism. J Hepatol. (2019) 70:237–48. doi: 10.1016/j.jhep.2018.10.033, PMID: 30658725 PMC6436537

[9] Tapper EB Parikh ND . Mortality due to cirrhosis and liver cancer in the United States, 1999–2016: observational study. BMJ. (2018) 362:k2817. doi: 10.1136/bmj.k2817, PMID: 30021785 PMC6050518

[10] Wang X Yu H Xing R Li P . Hepatoprotective effect of oyster peptide on alcohol-induced liver disease in mice. Int J Mol Sci. (2022) 23:8081. doi: 10.3390/ijms23158081, PMID: 35897657 PMC9332721

[11] Pei J Pan G Huang P Pushparaj R Zhang L Feng W . The effects of taurine on alcohol-associated liver disease are dose-dependent and associated with alterations of taurine-conjugated bile acids and FXR–FGF15 signaling. J Pharmacol Exp Ther. (2024) 389:408 (ASPET abstract). doi: 10.1124/jpet.408.131198, PMID: 35230691

[12] Lee HJ Saravana PS Ho TC Cho YJ Park JS Lee SG . *In vivo* protective effect against ethanol metabolism and liver injury of oyster (Crassostrea gigas) extracts obtained via subcritical water processing. Food Sci Biotechnol. (2021) 30:1063–74. doi: 10.1007/s10068-021-00941-9, PMID: 34471560 PMC8364599

[13] Liu C Miao Y Zhou W Ma Y Guo W Li A . Impact of thermal processing on the structure, antioxidant properties and hypoglycemic activities of sweet potato polysaccharides. Foods. (2024) 13:3082. doi: 10.3390/foods13193082, PMID: 39410117 PMC11475978

[14] Osaki K Shimizu Y Yamamoto T Miyake F Kondo S Yamaguchi H . Improvement of liver function by the administration of oyster extract as a dietary supplement to habitual alcohol drinkers: A pilot study. Exp Ther Med. (2015) 10:705–10. doi: 10.3892/etm.2015.2563, PMID: 26622379 PMC4509380

[15] Jiang S Ma Y Li Y Liu R Zeng M . Mediation of the microbiome-gut axis by oyster (*Crassostrea gigas*) polysaccharides: A possible protective role in alcoholic liver injury. Int J Biol Macromol. (2021) 182:968–76. doi: 10.1016/j.ijbiomac.2021.04.050, PMID: 33887288

[16] Shi X Ma H Tong C Qu M Jin Q Li W . Hepatoprotective effect of a polysaccharide from *Crassostrea gigas* on acute and chronic models of liver injury. Int J Biol Macromol. (2015) 78:142–8. doi: 10.1016/j.ijbiomac.2015.03.056, PMID: 25869310

[17] Zhang C Li X Jing X Zhang B Zhang Q Niu Q . Protective effects of oyster extract against hepatic tissue injury in alcoholic liver diseases. J Ocean Univ China. (2014) 13:262–70. doi: 10.1007/s11802-014-2449-0, PMID: 41816700

[18] Zhao G Zhai X Qu M Tong C Li W . Sulfated modification of the polysaccharides from Crassostrea gigas and their antioxidant and hepatoprotective activities through metabolomics analysis. Int J Biol Macromol. (2019) 129:386–95. doi: 10.1016/j.ijbiomac.2019.02.053, PMID: 30753880

[19] Siregar AS Nyiramana MM Kim EJ Shin EJ Woo MS Kim JM . Oyster broth concentrate and its major component taurine alleviate acute alcohol-induced liver damage. Food Sci Nutr. (2022) 10:2390–9. doi: 10.1002/fsn3.2847, PMID: 35844927 PMC9281932

[20] Byun JH Shin JE Choi YJ Choung SY . Oyster hydrolysate ameliorates ethanol diet-induced alcoholic fatty liver by regulating lipid metabolism in rats. Int J Food Sci Technol. (2021) 56:3556–66. doi: 10.1111/ijfs.14983, PMID: 41814451

[21] Gao S Shi J Wang K Tan Y Hong H Luo Y . Protective effects of oyster protein hydrolysates on alcohol-induced liver disease (ALD) in mice: based on the mechanism of anti-oxidative metabolism. Food Funct. (2022) 13:8411–24. doi: 10.1039/d2fo00660j, PMID: 35857308

[22] Wang K Shi J Gao S Hong H Tan Y Luo Y . Oyster protein hydrolysates alleviated chronic alcohol-induced liver injury in mice by regulating hepatic lipid metabolism and inflammation response. Food Res Int. (2022) 160:111647. doi: 10.1016/j.foodres.2022.111647, PMID: 36076379

[23] European Association for the Study of the Liver (EASL) . EASL Clinical Practice Guidelines: Management of alcohol-related liver disease. J Hepatol. (2018) 69:154–81. doi: 10.1016/j.jhep.2018.03.018, PMID: 29628280

[24] Crabb DW Im GY Szabo G Mellinger JL Lucey MR . Diagnosis and treatment of alcohol-associated liver diseases: 2019 Practice Guidance from the American Association for the Study of Liver Diseases. Hepatology. (2020) 71:306–33. doi: 10.1002/hep.30866, PMID: 31314133

[25] Daswani R Kumar A Sharma P Singla V Bansal N Arora A . Role of liver transplantation in severe alcoholic hepatitis. Clin Mol Hepatol. (2018) 24:43–50. doi: 10.3350/cmh.2017.0027, PMID: 29316778 PMC5875200

[26] Gawrieh S Dasarathy S Tu W Kamath PS Chalasani NP McClain CJ . AlcHepNet Investigators. Randomized trial of anakinra plus zinc vs. prednisone for severe alcohol-associated hepatitis. J Hepatol. (2024) 80:684–93. doi: 10.1016/j.jhep.2024.01.031, PMID: 38342441 PMC11214682

[27] Foretz M Even PC Viollet B . AMPK activation reduces hepatic lipid content by increasing fat oxidation *in vivo*. Int J Mol Sci. (2018) 19:2826. doi: 10.3390/ijms19092826, PMID: 30235785 PMC6164956

[28] Lu H-Y Zhao X Liu T-J Liang X Zhao M-Z Tian X-Y . Anti-obesity effect of fucoidan from Laminaria japonica and its hydrothermal degradation product. Food Biosci. (2024) 58:103749. doi: 10.1016/j.fbio.2024.103749, PMID: 41815951

[29] Oh RC Lanier JB . Management of hypertriglyceridemia. Am Fam Phys. (2007) 75:1365–71. doi: 10.1016/j.ijnurstu.2019.103422, PMID: 17508532

[30] Nguyen-Khac E Thevenot T Piquet M-A Benferhat S Goria O Chatelain D . Glucocorticoids plus N-acetylcysteine in severe alcoholic hepatitis. N Engl J Med. (2011) 365:1781–9. doi: 10.1056/NEJMoa1101214, PMID: 22070475

[31] Shasthry SM . Fecal microbiota transplantation in alcohol related liver diseases. Clin Mol Hepatol. (2020) 26:294–301. doi: 10.3350/cmh.2020.0057, PMID: 32570299 PMC7364360

[32] Kim D-h Sim Y Hwang J-h Kwun I-S Lim J-H Kim J . Ellagic acid prevents binge alcohol-induced leaky gut and liver injury through inhibiting gut dysbiosis and oxidative stress. Antioxidants. (2021) 10:1386. doi: 10.3390/antiox10091386, PMID: 34573017 PMC8465052

[33] Mi J Tong Y Zhang Q Wang Q Wang Y Wang Y . Alginate oligosaccharides enhance gut microbiota and intestinal barrier function, alleviating host damage induced by deoxynivalenol in mice. J Nutr. (2024) 154:3190–202. doi: 10.1016/j.tjnut.2024.09.031, PMID: 39357672

[34] Jung JH Kim SE Suk KT Kim DJ . Gut microbiota-modulating agents in alcoholic liver disease: Links between host metabolism and gut microbiota. Front Med (Lausanne). (2022) 9:913842. doi: 10.3389/fmed.2022.913842, PMID: 35935787 PMC9354621

[35] Chi X Sun X Cheng D Liu S Pan CQ Xing H . Intestinal microbiome-targeted therapies improve liver function in alcohol-related liver disease by restoring bifidobacteria: a systematic review and meta-analysis. Front Pharmacol. (2024) 14:1274261. doi: 10.3389/fphar.2023.1274261, PMID: 38259268 PMC10800551

[36] Rabelo ACS Andrade AKL Costa DC . The role of oxidative stress in alcoholic fatty liver disease: A systematic review and meta-analysis of preclinical studies. Nutrients. (2024) 16:1174. doi: 10.3390/nu16081174, PMID: 38674865 PMC11055095

