# Supplementary material for: Hepatoprotective effects of oyster-derived bioactive compounds in alcoholic liver disease: a systematic review
Source: Front Gastroenterol (Lausanne). 2026 Mar 17;5:1737942. doi: 10.3389/fgstr.2026.1737942 (PMC13035715; doi:10.3389/fgstr.2026.1737942)
Supplement: Supplementary file 1 [file DataSheet1.zip › supplementary/GOO250623-PROSPERO (2).pdf]

NIHR | National Institute for Health and Care Research

PROSPERO

International prospective register of systematic reviews

A systematic review on oyster polysaccharides in the prevention and treatment of alcoholic hepatitis: effects, mechanisms, and future prospects

Ping Yu, Rui Chen, Yanan Qin

Citation

Ping Yu, Rui Chen, Yanan Qin. A systematic review on oyster polysaccharides in the prevention and treatment of alcoholic hepatitis: effects, mechanisms, and future prospects. PROSPERO 2025 CRD420251104584. Available from https://www.crd.york.ac.uk/PROSPERO/view/CRD420251104584.

REVIEW TITLE AND BASIC DETAILS

Review title

A systematic review on oyster polysaccharides in the prevention and treatment of alcoholic hepatitis: effects, mechanisms, and future prospects

Condition or domain being studied

Alcoholic liver disease; Alcoholic Hepatitis

Alcoholic liver disease (ALD), including alcoholic hepatitis (AH)

ALD is a progressive liver condition caused by chronic excessive alcohol consumption. It includes a spectrum of liver injury ranging from steatosis to alcoholic hepatitis, fibrosis, cirrhosis, and hepatocellular carcinoma. This review focuses on ALD and AH as specific outcomes of interest in preclinical and clinical studies evaluating oyster polysaccharides.

Suggested PICO tags:

✅ Alcoholic Liver Disease

✅ Alcoholic Hepatitis

✅ Hepatoprotection

✅ Polysaccharides

✅ Nutritional Supplements

Rationale for the review

Oyster polysaccharides have shown promising antioxidant, anti-inflammatory, and hepatoprotective effects in experimental models of alcoholic liver disease (ALD), including alcoholic hepatitis (AH). These effects appear to target key mechanisms of ALD progression, such as oxidative stress, lipid peroxidation, and inflammatory cytokine production. Oysters are widely consumed as food, and their polysaccharides have been proposed as a potential natural supplement for liver health.

However, most of the existing evidence comes from scattered preclinical studies with varied designs, and the underlying mechanisms remain incompletely understood. Cooking methods, such as steaming, which are commonly used to prepare oysters, may also alter the structure and bioactivity of polysaccharides, potentially affecting their therapeutic value.

To date, no systematic review has synthesized the available evidence on oyster polysaccharides in ALD and AH. This review will fill this gap by summarizing and critically appraising current research on their effects, mechanisms of action, and translational potential. The findings may help clarify their role in liver disease prevention and treatment and guide future research and public health strategies.

Review objectives

To systematically review and critically appraise the current evidence on the effects of oyster polysaccharides in the prevention and treatment of alcoholic liver disease, including alcoholic hepatitis. Specifically, this review aims to:

Summarize the reported hepatoprotective effects of oyster polysaccharides in preclinical and clinical studies.

Explore the proposed mechanisms of action, including antioxidant, anti-inflammatory, and lipid-modulating pathways.

Assess the potential feasibility and implications of using oyster polysaccharides as a dietary supplement for liver health in populations at risk.

Keywords

oyster polysaccharides; Alcoholic liver disease; Alcoholic hepatitis; Hepatoprotective; Antioxidants

Country

China

ELIGIBILITY CRITERIA

Population

Included

Animal models of alcoholic liver disease or alcoholic hepatitis induced by ethanol.

In vitro studies using hepatocyte cell lines or primary hepatocytes exposed to ethanol.

Human participants diagnosed with alcoholic liver disease or alcoholic hepatitis, or with documented chronic alcohol consumption and liver injury.

Excluded

Studies of liver injury caused by agents other than alcohol (e.g., CCl_4, drugs, viruses).

Studies without any alcohol-induced injury model.

Studies on healthy populations without induced liver damage.

Intervention(s) or exposure(s)

Included

Nutritional supplement

Comparator(s) or control(s)

Included

PICO tags selected: Placebo; General Treatment; Supportive care

Studies must include a control group such as untreated, placebo, vehicle, usual care, or no intervention. Studies without a comparator group may still be included if they report relevant outcomes, but results will be interpreted accordingly.

Excluded

Comparators that involve other active interventions (e.g., drugs, supplements) where the effect of oyster polysaccharides cannot be isolated.

Study design

Both randomized and nonrandomized study types will be included.

Included

We will include randomized controlled trials, quasi-experimental studies, observational studies, controlled animal experiments, uncontrolled animal experiments, and controlled in vitro studies. Both preventive and therapeutic intervention studies will be considered.

Excluded

We will exclude narrative reviews, editorials, letters, case reports, and studies that do not include original data.

Context

This review includes studies conducted in laboratory, preclinical, and clinical settings. Animal studies carried out in controlled experimental facilities, in vitro studies using cultured hepatocytes, and human studies conducted in clinical or community settings are eligible. No restrictions will be placed on geographic location or healthcare setting.

TIMELINE OF THE REVIEW

Date of first submission to PROSPERO

16 July 2025

Review timeline

Start date: 20 June 2025. End date: 16 September 2025.

Date of registration in PROSPERO

21 July 2025

AVAILABILITY OF FULL PROTOCOL

Availability of full protocol

A full protocol has been written and uploaded to PROSPERO. The protocol will be made available after the review is completed.

SEARCHING AND SCREENING

Search for unpublished studies

Only published studies will be sought.

Main bibliographic databases that will be searched

The main databases to be searched are *CENTRAL - Cochrane Central Register of Controlled Trials*, *CLIB - The Cochrane Library*, *Embase.com*, *MEDLINE*, *PubMed* and *Scopus*.

Search language restrictions

The review will only include studies published in English.

Search date restrictions

Databases will be searched for articles published before 19 June 2025, there are no restrictions on search start date.

Other methods of identifying studies

Other studies will be identified by: *contacting authors or experts*, *looking through all the articles that cite the papers included in the review ("snowballing")*, *reference list checking*, *searching conference proceedings*, *searching dissertation and thesis databases* and *searching trial or study registers*.

Link to search strategy

A full search strategy has been uploaded to PROSPERO. The PDF may be accessed through this link https://www.crd.york.ac.uk/PROSPEROFILES/4b911760f0f0da95e1361411f4a7f23d.pdf.

Selection process

Studies will be screened independently by at least two people (or person/machine combination) with a process to resolve differences.

Other relevant information about searching and screening

We will manually screen the reference lists of all included studies and relevant reviews to identify additional eligible studies not captured in the database searches. Grey literature, such as theses and conference abstracts, will be identified through Google Scholar and institutional repositories where possible. If full texts of potentially eligible studies cannot be retrieved through institutional access, we will attempt to contact the authors directly. Screening of titles/abstracts and full texts will be carried out independently by two reviewers, with disagreements resolved through discussion or adjudication by a third reviewer.

DATA COLLECTION PROCESS

Data extraction from published articles and reports

Data will be extracted independently by at least two people (or person/machine combination) with a process to resolve differences.

Authors will be asked to provide any required data not available in published reports.

Study risk of bias or quality assessment

Risk of bias will be assessed using: *Cochrane RoB-1*, *Newcastle-Ottawa*

SYRCLE Risk of Bias

Data will be assessed independently by at least two people (or person/machine combination) with a process to resolve differences.

Additional information will be sought from study investigators if required information is unclear or unavailable in the study publications/reports.

Reporting bias assessment

We will assess risk of bias due to missing results (reporting bias) by visually inspecting funnel plots when at least 10 studies are included in a synthesis. Egger's test will also be performed to statistically evaluate asymmetry, if applicable.

Certainty assessment

We will use the GRADE approach to assess the certainty of evidence for each outcome, considering study limitations, consistency of results, directness of evidence, precision of estimates, and risk of publication bias. The certainty of evidence will be rated as high, moderate, low, or very low.

OUTCOMES TO BE ANALYSED

Main outcomes

The primary outcome is the effect of oyster polysaccharides on liver injury in alcoholic liver disease or alcoholic hepatitis models. This will include:

Biochemical markers of liver injury (e.g., serum alanine aminotransferase [ALT], aspartate aminotransferase [AST]), measured in IU/L.

Histological liver damage scores, assessed by validated scoring systems.

Oxidative stress markers, such as malondialdehyde (MDA), superoxide dismutase (SOD), and glutathione peroxidase (GPx) activity.

Inflammatory cytokine levels (e.g., TNF-α, IL-6, IL-1β), measured by ELISA or other immunoassays.

Outcomes will be reported at the latest time point measured in each study. Continuous outcomes will be summarized as mean differences or standardized mean differences with 95% confidence intervals if quantitative synthesis is feasible.

Additional outcomes

Changes in hepatic lipid content (e.g., triglycerides, cholesterol) measured in liver tissue or serum.

Changes in gut–liver axis indicators (e.g., gut microbiota composition, short-chain fatty acids), if reported.

Measures of fibrosis (e.g., collagen deposition, hydroxyproline content, fibrotic score).

Adverse events or reported toxicity outcomes (e.g., mortality, weight loss, abnormal organ findings).

Behavioral or clinical outcomes in human studies (e.g., quality of life, symptom scores).

PLANNED DATA SYNTHESIS

Strategy for data synthesis

We will conduct a formal quantitative synthesis (meta-analysis) if studies are sufficiently homogeneous in terms of populations, interventions, comparators, and outcomes. Continuous outcomes will be combined using mean differences or standardized mean differences with 95% confidence intervals, using a random-effects model. Heterogeneity will be assessed with the I^2 statistic, and potential publication bias will be evaluated with funnel plots and Egger's test if at least 10 studies contribute to an analysis.

If the included studies are too heterogeneous to allow meaningful quantitative synthesis, we will present the findings narratively, structured around the review objectives, and highlight consistent patterns where possible.

CURRENT REVIEW STAGE

Stage of the review at this submission

**Review stage**	**Started**	**Completed**
Pilot work	✓	
Formal searching/study identification	✓	
Screening search results against inclusion criteria	✓	

Review stage	Started	Completed
Data extraction or receipt of IPD		
Risk of bias/quality assessment		
Data synthesis		

Review status

The review is currently planned or ongoing.

Publication of review results

Results of the review will be published in English.

REVIEW AFFILIATION, FUNDING AND PEER REVIEW

Review team members

Mr Ping Yu (review guarantor and contact) Clinical Nutrition Department, Yantaishan Hospital, Yantai, Shandong, China. China.

No conflict of interest declared.

Mr Rui Chen. Clinical Nutrition Department, Yantaishan Hospital, Yantai, Shandong, China. China.

No conflict of interest declared.

Mr Yanan Qin. Women's Health Department, Zhaoyuan Maternal and Child Health Hospital, Yantai, Shandong, China. China.

No conflict of interest declared.

Named contact

Mr Ping Yu (pingyu_@outlook.com). Clinical Nutrition Department, Yantaishan Hospital, Yantai, Shandong, China. China.

Review affiliation

Clinical Nutrition Department, Yantaishan Hospital, Yantai, Shandong, China

Funding source

Review has no funding and no agreed support from an academic institution and is done in authors’ own time.

Peer review

There has been no peer review of this planned review.

ADDITIONAL INFORMATION

Review conflict of interest

Declared individual interests are recorded under team member details.. No additional interests are recorded for this review.

Medical Subject Headings

Alcohol Drinking; Anti-Inflammatory Agents; Antioxidants; Carcinoma, Hepatocellular; Dietary Supplements; Hepatitis; Hepatitis, Alcoholic; Humans; Lipids; Liver Cirrhosis, Alcoholic; Liver Diseases, Alcoholic; Liver Neoplasms; Risk Factors; Crassostrea; Polysaccharides

SIMILAR REVIEWS

Check for similar records already in PROSPERO

PROSPERO identified a number of existing PROSPERO records that were similar to this one (last check made on 15 July 2025). These are shown below along with the reasons given by that the review team for the reviews being different and/or proceeding.

- Effect of Intestinal Microbiome-targeted Therapies in Treatment of Alcohol-related Liver Disease: a systematic review and meta-analysis [published 18 June 2021] [CRD42021246067]. The review was judged **not to be similar**
- Effect of obesity on severity and mortality among patients with alcoholic liver diseases: a systematic review and meta-analysis [published 9 February 2022] [CRD42022300673]. The review was judged **not to be similar**
- Evidence construction of Silibinin capsules against alcoholic liver disease based on a systematic review and network pharmacology [published 23 February 2024] [CRD42024509676]. The review was judged **not to be similar**

PROSPERO version history

- Version 1.0, published 21 Jul 2025

Disclaimer

The content of this record displays the information provided by the review team. PROSPERO does not peer review registration records or endorse their content.

PROSPERO accepts and posts the information provided in good faith; responsibility for record content rests with the review team. The guarantor for this record has affirmed that the information provided is truthful and that they understand that deliberate provision of inaccurate information may be construed as scientific misconduct.

PROSPERO does not accept any liability for the content provided in this record or for its use. Readers use the information provided in this record at their own risk.

Any enquiries about the record should be referred to the named review contact
