# Supplementary material for: Hepatoprotective effects of oyster-derived bioactive compounds in alcoholic liver disease: a systematic review
Source: Front Gastroenterol (Lausanne). 2026 Mar 17;5:1737942. doi: 10.3389/fgstr.2026.1737942 (PMC13035715; doi:10.3389/fgstr.2026.1737942)
Supplement: Supplementary file 1 [file DataSheet1.zip › supplementary/Prisma guideline completed.docx]

Here is the filled-up PRISMA 2020 checklist with approximate page numbers from the manuscript for each section/location where the item is reported:

| Section and Topic | Item # | Checklist item | Location where item is reported (page no) |
| --- | --- | --- | --- |
| TITLE | 1 | Identify the report as a systematic review. | 1 |
| ABSTRACT | 2 | See the PRISMA 2020 for Abstracts checklist. | 1-2 |
| INTRODUCTION | 3 | Describe the rationale for the review in the context of existing knowledge. | 2-3 |
|  | 4 | Provide an explicit statement of the objective(s) or question(s) the review addresses. | 3 |
| METHODS | 5 | Specify the inclusion and exclusion criteria for the review and how studies were grouped. | 4 |
|  | 6 | Specify all databases, registers, websites, organisations, reference lists searched/consulted. | 4-5 |
|  | 7 | Present the full search strategies for all databases, including filters and limits used. | 4-5 |
|  | 8 | Specify the methods used to decide whether a study met the inclusion criteria. | 6 |
|  | 9 | Specify methods used to collect data from reports, number of reviewers, independence, etc. | 5 |
|  | 10a | List and define all outcomes for which data were sought. | 4-6 |
|  | 10b | List and define other variables for which data were sought; assumptions about missing info. | 4-6 |
|  | 11 | Specify methods used to assess risk of bias in included studies. | 6-7 |
|  | 12 | Specify effect measures used in the synthesis or presentation of results. | 4-6 |
|  | 13a-f | Describe methods to decide which studies eligible, data preparation, display, synthesis etc. | 4-7 |
|  | 14 | Describe methods used to assess risk of bias due to missing results. | 6-7 |
|  | 15 | Describe methods used to assess certainty in the body of evidence. | 7 |
| RESULTS | 16a | Describe results of search and selection process, ideally with flow diagram. | 7-8,23 |
|  | 16b | Cite studies excluded that might appear to meet inclusion criteria and explain why. | 9-11 |
|  | 17 | Cite each included study and present characteristics. | 9-15 |
|  | 18 | Present assessments of risk of bias for each included study. | 24-25 |
|  | 19 | Present, for each study, summary statistics and effect estimates with precision. | 15-23 |
|  | 20a-d | For each synthesis, summarise characteristics and risk of bias; results and heterogeneity. | 24-25 |
|  | 21 | Present assessments of risk of bias due to missing results. | 25 |
|  | 22 | Present assessments of certainty in the body of evidence. | 25-27 |
| DISCUSSION | 23a | Provide general interpretation of results in context of other evidence. | 27-28 |
|  | 23b | Discuss any limitations of evidence included. | 30 |
|  | 23c | Discuss any limitations of the review processes used. | 30 |
|  | 23d | Discuss implications of results for practice, policy, and future research. | 29-30 |
| OTHER INFORMATION | 24a | Provide registration information including register name and registration number. | 3-4 |
|  | 24b | Indicate where review protocol can be accessed or state that it was not prepared. | 32 |
|  | 24c | Describe and explain any amendments to information at registration or protocol. | 32 |
|  | 25 | Describe sources of financial/non-financial support and role of funders/sponsors. | 31 |
|  | 26 | Declare any competing interests of review authors. | 31 |
|  | 27 | Report which data, code, and materials are publicly available and where. | 32 |
