# Supplementary material for: Hepatoprotective effects of oyster-derived bioactive compounds in alcoholic liver disease: a systematic review
Source: Front Gastroenterol (Lausanne). 2026 Mar 17;5:1737942. doi: 10.3389/fgstr.2026.1737942 (PMC13035715; doi:10.3389/fgstr.2026.1737942)
Supplement: Supplementary file 1 [file DataSheet1.zip › supplementary/Supplementary Material S1.docx]

# **Supplementary Material S1. Full Search Strategies**

### **PubMed**

(

"Liver Diseases, Alcoholic"[Mesh] OR

"alcoholic liver disease"[TIAB] OR

"alcohol-related liver disease"[TIAB] OR

"alcohol-induced liver injury"[TIAB] OR

"alcoholic hepatitis"[TIAB] OR

"alcoholic steatosis"[TIAB] OR

"alcoholic fatty liver"[TIAB] OR

"alcoholic fibrosis"[TIAB] OR

"alcoholic cirrhosis"[TIAB] OR

"ethanol-induced hepatotoxicity"[TIAB] OR

"alcohol hepatotoxicity"[TIAB] OR

hepat* AND alcohol*[TIAB]

)

AND

(

"Crassostrea"[Mesh] OR

"Polysaccharides"[Mesh] OR

oyster*[TIAB] OR

Crassostrea*[TIAB] OR

"oyster polysaccharide*"[TIAB] OR

"oyster peptide*"[TIAB] OR

"oyster hydrolysate*"[TIAB] OR

"oyster extract*"[TIAB] OR

"oyster protein*"[TIAB] OR

"marine polysaccharide*"[TIAB] OR

bivalve*[TIAB] OR

"shellfish extract*"[TIAB]

)

Filters: none applied (all years to June 30, 2024).
 Field tags: [TIAB] = Title/Abstract.

### **Web of Science Core Collection**

TS=(

"alcoholic liver disease" OR

"alcohol-related liver disease" OR

"alcohol-induced liver injury" OR

"alcoholic hepatitis" OR

"alcoholic steatosis" OR

"alcoholic fatty liver" OR

"alcoholic fibrosis" OR

"alcoholic cirrhosis" OR

"ethanol-induced hepatotoxicity" OR

"alcohol hepatotoxicity" OR

(alcohol* NEAR/3 hepat*)

)

AND

TS=(

oyster* OR

Crassostrea* OR

"oyster polysaccharide*" OR

"oyster peptide*" OR

"oyster hydrolysate*" OR

"oyster extract*" OR

"oyster protein*" OR

"marine polysaccharide*" OR

bivalve* OR

"shellfish extract*"

)

Timespan: Inception – June 30, 2024.
 Indexes: SCI-EXPANDED, SSCI, A&HCI, ESCI.

### **Scopus**

(TITLE-ABS-KEY(

"alcoholic liver disease" OR

"alcohol-related liver disease" OR

"alcohol-induced liver injury" OR

"alcoholic hepatitis" OR

"alcoholic steatosis" OR

"alcoholic fatty liver" OR

"alcoholic fibrosis" OR

"alcoholic cirrhosis" OR

"ethanol-induced hepatotoxicity" OR

"alcohol hepatotoxicity" OR

(alcohol* W/3 hepat*)

))

AND

(TITLE-ABS-KEY(

oyster* OR

Crassostrea* OR

"oyster polysaccharide*" OR

"oyster peptide*" OR

"oyster hydrolysate*" OR

"oyster extract*" OR

"oyster protein*" OR

"marine polysaccharide*" OR

bivalve* OR

"shellfish extract*"

))

Search fields: Title, Abstract, Keywords.
 Language: No restriction (translations handled during screening).

### **Grey Literature & Additional Sources**

- **Clinical trial registries**: ClinicalTrials.gov, WHO ICTRP searched with: *“oyster extract” AND “alcoholic liver disease”*.
- **Conference abstracts & dissertations**: ProQuest Dissertations & Theses, OpenGrey, and relevant conference proceedings (Asian Pacific Association for the Study of the Liver).
- **Backward and forward citation searching**: Performed in Web of Science and Scopus for all included articles.
- **Hand-searching**: Hepatology, Journal of Hepatology, Alcohol and Alcoholism, Marine Drugs (last 10 years).
