# Supplementary material for: Hepatoprotective effects of oyster-derived bioactive compounds in alcoholic liver disease: a systematic review
Source: Front Gastroenterol (Lausanne). 2026 Mar 17;5:1737942. doi: 10.3389/fgstr.2026.1737942 (PMC13035715; doi:10.3389/fgstr.2026.1737942)
Supplement: Supplementary file 1 [file DataSheet1.zip › supplementary/Supplementary Table S1.docx]

Supplementary Table S1. Detailed methodological characteristics of included studies investigating oyster-derived bioactives for alcohol-related liver disease

| **Author(s), year** | **Title** | **Country** | **Study Type** | **Inclusion Criteria** | **Exclusion Criteria** | **Model Type** | **Duration of Intervention** | **Study Population Characteristics** | **Control Group** | **Randomization / Blinding** |
| --- | --- | --- | --- | --- | --- | --- | --- | --- | --- | --- |
| Osaki et al., 2015 | Improvement of liver function by the administration of oyster extract as a dietary supplement to habitual alcohol drinkers: A pilot study | Japan | Randomized, double-blind, placebo-controlled feeding trial | Japanese adults aged 20-65 years, alcohol drinking 5-7 times/week, GGT 50-150 IU/l, BMI 18-30 kg/m² | Medications affecting alcohol metabolism, current OE consumption, hepatitis virus positive, pregnancy/breastfeeding, significant medical conditions | Human clinical trial | 12 weeks | N=84 enrolled (42 per group), 74 completed efficacy analysis (38 placebo, 36 OE). Age: Placebo 48.8±9.1 years, OE 46.3±8.7 years. Gender: 31 males, 11 females per group. BMI: Placebo 23.8±2.9, OE 24.3±3.2 kg/m² | Yes - placebo group (n=42, matched for demographics) | Randomized (1:1 ratio using random number tables), double-blind, placebo-controlled |
| Jiang et al., 2021 | Mediation of the microbiome-gut axis by oyster (Crassostrea gigas) polysaccharides: A possible protective role in alcoholic liver injury | China (Ocean University of China, Qingdao) | Experimental animal study | 8-week-old male C57BL/6 mice, weighing 20-22g, healthy laboratory animals | Not explicitly stated for animal model | Animal model (C57BL/6 mice) | 4 weeks polysaccharide treatment + 28 days ethanol feeding (total ~5 weeks) | N=40 total mice (10 per group): Control, EtOH (ethanol model), EtOH+RPS (raw polysaccharides), EtOH+SPS (steamed polysaccharides). Standard laboratory conditions (12-h light:dark cycle, 22±2°C, 55±10% humidity) | Yes - control group fed Lieber-DeCarli diet without ethanol | Randomized into 4 groups (n=10 each), not blinded |
| Shi et al., (2015) | Hepatoprotective effect of a polysaccharide from Crassostrea gigas on acute and chronic models of liver injury | China (Dalian Ocean University) | Animal experimental study - in vivo controlled trial using laboratory mice | 6-8 week old BALB/c mice of either sex for ethanol model; 7-week old C57BL/6J mice of either sex for CCl4 model; purchased from Laboratory Animal Center of Dalian Medical University | Not explicitly stated in the methodology | Dual model system: (1) Chronic ethanol-induced hepatotoxicity model using BALB/c mice, (2) Acute CCl4-induced liver injury model using C57BL/6J mice | 14 days of continuous treatment for both acute and chronic models | Healthy laboratory mice maintained under standard husbandry conditions: 22±2°C temperature, 60-70% relative humidity, 12-hour light-dark cycle, free access to sterile standard mouse chow and water, well-ventilated housing | Multiple controls: (1) Saline-treated negative control group, (2) Positive control groups - DDB (200 mg/kg) for CCl4 model, Tiopronin (50 mg/kg) for ethanol model | Randomized allocation into 6 groups per model (n=8 animals per group), blinding status not specified in methodology |
| Zhu et al., 2023 | Polysaccharides from Ostrea rivularis rebuild the balance of gut microbiota to ameliorate non-alcoholic fatty liver disease in ApoE−/− mice | China (Guangdong Academy of Sciences, Guangzhou University of Chinese Medicine) | Animal experimental study - in vivo controlled trial using genetically modified mice | Male ApoE−/− mice, 8 weeks old, 22±2g body weight, animal certificate number SCXK (Jing) 2014-0004, purchased from Beijing Huafukang Bioscience Co., LTD | Not explicitly stated in methodology | Non-alcoholic fatty liver disease (NAFLD) model induced by high-fat diet in ApoE−/− mice | 12 weeks of continuous intervention | Male apolipoprotein E knockout (ApoE−/−) mice housed in independent ventilation cages at Experimental Animal Center, Guangzhou University of Chinese Medicine; breeding environment: 22±2°C, 60-70% relative humidity, 12/12h light-dark cycle, standard food and water ad libitum | Multiple controls: (1) Normal group (NG) with normal diet, (2) Positive drug group (PDG) with Atorvastatin 5mg/kg as standard treatment | Randomized allocation into 5 groups according to body weight (n=8 mice per group), blinding status not specified |
| Zhang et al., 2014 | Protective Effects of Oyster Extract Against Hepatic Tissue Injury in Alcoholic Liver Diseases | China - Department of Digestive Diseases, Affiliated Hospital of Medical College, Qingdao University, Qingdao 266003 | Experimental animal study - Randomized controlled trial using Wistar rat model | Healthy male Wistar rats weighing 232.8±19.9g purchased from Experimental Animal Center of Tongji Medical of Huazhong University of Science & Technology | Not explicitly mentioned in the study protocol | Alcoholic liver disease (ALD) rat model induced by intragastric alcohol administration with progressive dose escalation | Total 9 weeks: 8 weeks of treatment (alcohol induction concurrent with oyster extract treatment) + 1 week normal feeding period before sacrifice and analysis | 75 male Wistar rats housed under controlled conditions (12h light/dark cycles, 23±2°C temperature), fed commercial standard rat chow and water ad libitum | Group 2: Healthy control rats (n=15) received distilled water for 8 weeks, no alcohol or treatment administration | Randomized allocation into 5 groups with 15 rats each. Blinding procedures not explicitly mentioned in methodology |
| Zhao et al., 2019 | Sulfated modification of the polysaccharides from Crassostrea gigas and their antioxidant and hepatoprotective activities through metabolomics analysis | China - College of Food Science and Engineering, Dalian Ocean University, Dalian 116023; Nutrition Department, The Second Hospital of Dalian Medical University | Experimental animal study with metabolomics analysis - controlled trial using Kunming mice model | Male Kunming mice (SPF grade, 18-22g) from Laboratory Animal Center of Dalian Medical University | Not explicitly mentioned in study protocol | Alcoholic liver injury mouse model induced by intragastric ethanol administration | Total 35 days: 15 days ethanol-induced liver injury + 20 days concurrent treatment period | 40 male Kunming mice housed in standard laboratory conditions (temperature 23±2°C, humidity 50-60%, 12h light-dark cycle), fed standard diet and water ad | Group 1: Negative control mice treated with saline (n=8) without ethanol or treatment | Mice randomly divided into 5 groups (n=8 each). Blinding procedures not mentioned in methodology |
| Lee et al., 2021 | In vivo protective effect against ethanol metabolism and liver injury of oyster (Crassostrea Gigas) extracts obtained via subcritical water processing | Republic of Korea | Experimental animal study (in vivo) | Male ICR mice, 5 weeks old, purchased from Sam Taco Bio Korea (Seongnam, Korea) | Not explicitly mentioned in study protocol | D-galactosamine (D-GalN)-induced acute liver injury model and acute ethanol intoxication model | 7 days pretreatment for D-GalN study; single dose 30 minutes before ethanol for alcohol metabolism study | Male ICR mice, 5 weeks old, maintained at 24.7 ± 0.5°C, humidity 53.0 ± 1.7%, fed AIN-93G diet during adaptation period | Normal saline (0.9%) for D-GalN study; distilled water for alcohol metabolism study | Mice randomly divided into groups; blinding not specified |
| Wang et al., 2022 | Oyster protein hydrolysates alleviated chronic alcohol-induced liver injury in mice by regulating hepatic lipid metabolism and inflammation response | China | Animal experimental study | Male C57BL/6J mice, aged 6-8 weeks, weighing 22.2 ± 0.08 g | Not specified | Chronic alcoholic liver disease mouse model | 9 weeks total (1 week adaptation + 8 weeks treatment) | Male C57BL/6J mice, n=12 per group, 6 groups total | Normal control group (Lindros liquid control diet for 9 weeks) | Randomized into 6 groups, blinding not specified |
| Wang et al., 2022 (2) | Hepatoprotective Effect of Oyster Peptide on Alcohol-Induced Liver Disease in Mice | China | Animal experimental study | C57BL/6 mice, 18-22g, 4-week-old | Not specified | Chronic alcoholic liver disease mouse model | 6 weeks treatment | Male C57BL/6 mice, n=10 per group (after dropouts), 6 groups total | Control group (physiological saline 10 mL/kg bw daily) | Randomized into 6 groups, blinding not specified |
| Siregar et al., 2022 | Oyster broth concentrate and its major component taurine alleviate acute alcohol-induced liver damage | Korea | Original Research (Animal Study) | Male C57BL/6 mice (7 weeks old), maintained under 12-h light/dark cycle, specific pathogen-free facility, food and water freely available for 1 week prior to experiment | Not explicitly stated | Single-EtOH-binge mouse model | 7 hours post-EtOH administration | Male C57BL/6 mice (7 weeks old), n=15 per group for behavioral tests, n=12 for enzyme assays, n=5 for histology, n=6 for cytokine analysis, n=3 for western blots | Vehicle (saline + saline) | Randomized into 5 groups (vehicle, EtOH+saline, EtOH+OBC, EtOH+OH, EtOH+taurine), no blinding mentioned |
| Byun et al., 2021 | Oyster hydrolysate ameliorates ethanol diet-induced alcoholic fatty liver by regulating lipid metabolism in rats | Korea | Original Research (Animal Study) | Male Sprague-Dawley rats (5 weeks old), housed in standard animal facility with 12-h:12-h light-dark cycle at 25±1°C, free access to food and water | Not explicitly stated | Chronic ethanol diet-induced alcoholic fatty liver rat model | 10 weeks total (6 weeks ethanol diet + 4 weeks treatment) | Male SD rats (5 weeks old), n=10 per group, purchased from Central Lab. Animal Inc. (Seoul, Korea) | Normal diet group (ND) - liquid diet containing dextrin-maltose to match calories in ethanol | Randomly divided into groups, no mention of blinding |
| Gao et al., 2022 | Protective effects of oyster protein hydrolysates on alcohol-induced liver disease (ALD) in mice: based on the mechanism of anti-oxidative metabolism | China | In vivo experimental | Male C57BL/6 mice, 6-8 weeks old, weighing 22.2±0.08g | Not specified | C57BL/6 mice | 4 weeks | n=60 total (n=10 per group), 6 groups | Normal (no ethanol), Model (ethanol only), Positive control (silymarin 100mg/kg) | Randomly divided into groups, blinding not specified |
