# Supplementary material for: Hepatoprotective effects of oyster-derived bioactive compounds in alcoholic liver disease: a systematic review
Source: Front Gastroenterol (Lausanne). 2026 Mar 17;5:1737942. doi: 10.3389/fgstr.2026.1737942 (PMC13035715; doi:10.3389/fgstr.2026.1737942)
Supplement: Supplementary file 1 [file DataSheet1.zip › supplementary/Supplementary table S2.docx]

Supplementary Table S2: Comprehensive extraction methodologies, molecular characterizations (such as molecular weight, monosaccharide content, and degree of sulfation), and co-inducing variables.

| **Author(s), year** | **Oyster Species** | **Extract Type** | **Extraction Method** | **Bioactive Type** | **Dose Administered** | **Route of Administration** | **Frequency & Duration** | **ALD Induction Method** | **Ethanol Dose & Duration** | **Co-inducing Agents** |
| --- | --- | --- | --- | --- | --- | --- | --- | --- | --- | --- |
| Osaki et al., 2015 | Crassostrea gigas | Powdered oyster extract | Biochemical preparation process (specific method not detailed) | Glycogen (>50% w/w of the extract) | 1,000 mg oyster extract per day (333 mg per tablet × 3 tablets) | Oral (tablets) | 3 tablets daily before evening meal for 12 weeks | Natural - habitual alcohol drinkers (5-7 times per week) | Self-reported: Placebo group 53±27 g/day, OE group 47±25 g/day throughout study | None |
| Jiang et al., 2021 | Crassostrea gigas | Raw polysaccharides (RPS) and steamed polysaccharides | Hot water extraction, ultrasonic pretreatment (876W, 15min), enzymatic digestion (Alcalase 3000U/g, 55°C, 4h), alcohol precipitation, purification with trypsin/pepsin | RPS: 78.79% glucose, 14.31% rhamnose, 6.48% galacturonic acid, 4.1% mannose. SPS: Similar composition. Both high molecular weight (RPS: 8.2×10⁷ Da, SPS: 9.6×10⁷ Da) | 282 mg/kg body weight (equivalent to 50g fresh oyster meat for 70kg adult) | Intragastric gavage | Daily for 4 weeks during ethanol treatment period | Lieber-DeCarli liquid diet with gradual ethanol introduction | Ethanol concentration gradually increased from 1% to 5% (v/v) over 5 days, then maintained at 5% for 28 days. Additional acute ethanol gavage (31.5% v/v, volume = body weight×20μL) on days 11 and 21 | Isocaloric dextrin maltose as control for acute gavage |
| Shi et al., (2015) | Crassostrea gigas (Pacific oyster) collected from Dalian coast, Yellow Sea, April 2013; shells manually removed, cleaned and freeze-dried prior to extraction | Water-soluble polysaccharide designated as CGPS-1; appeared as white-yellow powder; no protein or nucleic acid contamination (no absorption at 280nm and 260nm) | Novel ethanol-free extraction process: Hot-water extraction (1:40 w/v ratio, 90-100°C), isoelectric precipitation (pH 5.0 for 12h, then pH 2.0), pepsin hydrolysis (2h), hollow fiber ultrafiltration (10 kDa cut-off polysulfone membrane, 0.25 m² effective area), freeze-drying | Uniform glucose polymer with β-configuration d-glucan structure; molecular weight ~6.5×10⁶ Da determined by TSK G4000PWXL column chromatography; composed exclusively of glucose monomers confirmed by PMP-derivatized HPLC analysis; yield 12.3% of dry oyster weight | Ethanol model: 50, 150, 450 mg/kg body weight; CCl4 model: 200, 400, 800 mg/kg body weight; doses selected based on preliminary toxicity screening | Oral administration (per os, p.o.) via gavage | Daily administration for 14 consecutive days in both models; treatment given before hepatotoxic agent administration | Chronic ethanol administration to induce alcoholic liver disease-like pathology in BALB/c mice | 50% (v/v) ethanol solution, 5 ml/kg body weight, administered orally daily for 14 days concurrent with CGPS-1 treatment | None for ethanol model; separate acute model used CCl4 (1.2 ml/kg body weight on day 14) as hepatotoxin |
| Zhu et al., 2023 | Ostrea rivularis (a kind of oyster distributed along the southeast coast of China); polysaccharide obtained and structurally characterized in previous reports [21] | Ostrea rivularis polysaccharide (ORP) - purified polysaccharide with previously characterized structure capable of activating Keap1-Nrf2/ARE pathway for antioxidant activity | Previously established extraction and purification method, specific extraction details not provided in current study but refers to prior methodology | Polysaccharide with antioxidant properties through Keap1-Nrf2/ARE pathway activation; specific structural details referenced from previous characterization studies | Low-dose group (LDG): 100 mg/kg body weight; High-dose group (HDG): 400 mg/kg body weight; doses selected based on preliminary studies | Oral administration via gavage | Daily administration for 12 consecutive weeks; FITC-dextran (1 mg/ml) administered 4h before sacrifice for intestinal permeability assessment | High-fat diet (HFD) induced NAFLD in ApoE−/− mice model; detailed feed compositions provided in Supplementary Table 1 | Not applicable - this is NAFLD model, not alcoholic liver disease; no ethanol administration | High-fat diet composition as co-inducing factor for NAFLD development in genetically susceptible ApoE−/− mice |
| Zhang et al., 2014 | Not specifically identified - extracted from high-quality oyster, scallop, and surf clam from Jiao Zhou Bay, China | Oyster extract (taurine ganbo) provided by Qingdao Dongyi Science and Technology Development Company, batch number QS370222020116, formulated as 0.4g/granule. The oyster extract was formulated as 0.4 g per granule and is reported to contain bioactive polysaccharides along with taurine, contributing to its hepatoprotective properties. | Zymohydrolysis extraction process from high-quality marine mollusks (oyster, scallop, surf clam), retaining high purity with no reported side effects | Not specifically characterized - contains rich glycogen, taurine, vitamin B, microelement zinc, essential amino acids, listed on National Health Ministry's medicine-food homology list | Group 3 (Low-dose): 0.12 g/kg/day; Group 4 (Middle-dose): 0.40 g/kg/day; Group 5 (High-dose): 1.20 g/kg/day | Not explicitly stated (presumed oral gavage based on dosing regimen and concurrent alcohol administration) | Daily administration for 8 consecutive weeks, with all groups fed normally for an additional week before sacrifice | Intragastric administration of 50% dehydrated alcohol (analytical pure grade) via stomach injection | Progressive dose escalation: Week 1-2: 8 mL/kg/day; Week 3-4: 10 mL/kg/day; Week 5-8: 15 mL/kg/day | None - alcohol was the sole hepatotoxic agent used |
| Zhao et al., 2019 | Crassostrea gigas (Pacific oyster) - commonly consumed seafood, widely cultured in Pacific coast of Asia | Sulfated polysaccharides from Crassostrea gigas (SCGP) prepared by chlorosulfonic acid-pyridine method with degree of substitution (DS) = 0.66 and sulfur content = 28.96% | Multi-step process: Hot water extraction → Sevag deproteinization (trichloromethane:n-butanol 5:1) → CAS-pyridine sulfation (60°C, 3h) → dialysis → lyophilization | Sulfated polysaccharides with molecular weight decreased from 6.5×10⁶ Da (CGP) to 3.3×10⁶ Da (SCGP), sulfate groups primarily substituted at C-6 position confirmed by ¹³C NMR | Low dose: 100 mg/kg body weight; Medium dose: 200 mg/kg body weight; High dose: 400 mg/kg body weight | Oral administration (intragastric) - route specifically mentioned for ethanol, presumed same for SCGP treatment | Daily administration during treatment period (concurrent with ethanol-induced injury for 15 days, then continued for additional 20 days) | Intragastric administration of ethanol to normal mice to induce alcoholic liver injury | 10 mL/kg body weight ethanol administered once daily for consecutive 15 days | None - ethanol was the sole hepatotoxic agent used for liver injury induction |
| Lee et al., 2021 | Crassostrea gigas (Pacific oyster) obtained from oyster farm in Yongnam-myeon, Tongyeong-si, Gyeongsangnam-do | Raw oyster lyophilized powder (OP) and subcritical water treated oyster powder (SOP) | Subcritical water extraction at 175°C, 60 bar pressure, 150 rpm for 5 minutes using 200 cm³ batch reactor; samples freeze-dried at -110°C for 48h, crushed to 710 μm | Glucose-based polysaccharides; OP contained maltose (17.329 ± 0.125 mg/g) and glucose (17.038 ± 0.238 mg/g); SOP contained only glucose (4.453 ± 0.211 mg/g). The subcritical water oyster extract powder (SOP) prepared from Crassostrea gigas using pressurized hot water extraction was confirmed to contain polysaccharides, as demonstrated by HPLC-based monosaccharide composition profiling, including glucose, galactose, arabinose, and mannose. | 250 mg/kg body weight (selected from dose-finding study testing 100, 250, 500 mg/kg) | Oral administration | Daily for 7 days (D-GalN study); single administration (alcohol metabolism study) | D-galactosamine (D-GalN) 850 mg/kg administered intraperitoneally 1 hour after final oral dose | 20% ethanol, 8 mL/kg body weight, single administration 30 minutes after sample administration | None specified |
| Wang et al., 2022 | Crassostrea gigas | Protein hydrolysate (OPH) | pH-shifting procedure + alcalase hydrolysis at 55°C for 2h (pH 8.0) | Peptides (85.65% <2kDa MW), mainly from myosin heavy chain (36.43%), myosin light chain (18.99%) | Low: 200 mg/kg, Medium: 400 mg/kg, High: 800 mg/kg | Intragastric gavage | Once daily for 8 weeks | Lindros ethanol diet | 5% ethanol diet for 8 weeks (after 1 week adaptation) | None |
| Wang et al., 2022 (2) | Crassostrea talienwhanensis | Oyster peptide (OP, MW <3500 Da) | Trypsin hydrolysis at pH 8.3, 41.7°C for 6.7h, ultrafiltration <3500 Da | 13 peptides rich in Lys, Arg, His, Thr | Low: 120 mg/kg, Medium: 240 mg/kg, High: 480 mg/kg | Oral gavage | Once daily for 6 weeks | 50% ethanol oral gavage | 10 mL/kg bw daily for 6 weeks | None |
| Siregar et al., 2022 | Crassostrea gigas | Oyster Broth Concentrate (OBC) + Taurine comparison | Boiling oysters at 95°C for 3 min, concentrated to brix 45.00 ± 0.16% | Taurine (41.8% of total free amino acids in OBC), plus glycine, proline, alanine, arginine, glutamate, carnosine dipeptide | OBC: 200 mg/kg; Taurine: 45 mg/kg; OH: 200 mg/kg (positive control) | Oral gavage | Single preadministration 1h before EtOH administration | Single-EtOH-binge administration | 3 g/kg EtOH, single dose, samples collected at 7h post-administration | None |
| Byun et al., 2021 | Crassostrea gigas (implied from previous studies) | TGPN (Transglutaminase, Protamex and Neutrase oyster hydrolysate) | Enzymatic hydrolysis using transglutaminase, protamex and neutrase enzymes | Tyrosine-alanine (YA) dipeptide as marker compound, with antioxidant, anti-inflammatory and antihypertensive effects | TGPN 50: 50 mg/kg; TGPN 100: 100 mg/kg; TGPN 200: 200 mg/kg; Silymarin 100: 100 mg/kg (positive control) | Oral administration | Daily for 4 weeks after 6 weeks of ethanol feeding | Lieber-DeCarli ethanol liquid diet method | 5% (v/v) ethanol diet (36% of energy content), gradually increased from 0% to 5% in first week, then 5% for 6 weeks | None |
| Gao et al., 2022 | Crassostrea gigas | Protein hydrolysates (AOPH) | Alcalase enzymatic hydrolysis (2h, pH 8.0, 55°C) | Small molecular peptides (<2kDa, 90.85%) | Low: 200mg/kg, Medium: 400mg/kg, High: 800mg/kg | Oral gavage | Once daily for 4 weeks | Lindros liquid control diet | 5% ethanol for 4 weeks (gradual introduction over 1 week) | None |
