# Supplementary material for: Hepatoprotective effects of oyster-derived bioactive compounds in alcoholic liver disease: a systematic review
Source: Front Gastroenterol (Lausanne). 2026 Mar 17;5:1737942. doi: 10.3389/fgstr.2026.1737942 (PMC13035715; doi:10.3389/fgstr.2026.1737942)
Supplement: Supplementary file 1 [file DataSheet1.zip › supplementary/Supplementary Table S3.docx]

Supplementary table S3: Primary Therapeutic Outcomes: Liver Function Enzymes, Lipid Metabolism, Oxidative Stress Markers, Inflammatory Response and Histological Changes Parameters

| **Author(s), year** | **Liver Enzymes Measured (ALT, AST, etc.)** | **Lipid Profile (TG, TC, etc.)** | **Oxidative Stress Markers (GSH, MDA, etc.)** | **Inflammatory Markers (TNF-α, IL-6, etc.)** | **Histological Changes** |
| --- | --- | --- | --- | --- | --- |
| Osaki et al., 2015 | GGT (primary): Significant between-group difference at week 12 (p=0.049). Placebo group increased 12% (79.3→88.5 IU/l), OE group decreased 8% (76.9→70.6 IU/l). ALT: Placebo increased 9% (28.7→31.4 IU/l), OE decreased 10% (31.0→27.5 IU/l) - not significant. AST: Placebo increased 2% (25.9→26.4 IU/l), OE decreased 10% (25.5→22.9 IU/l) - not significant | Baseline measurements only: LDL-cholesterol: Placebo 124±38 mg/dl, OE 129±43 mg/dl (p=0.570). HDL-cholesterol: Placebo 65±18 mg/dl, OE 64±17 mg/dl (p=0.684). Triglycerides: Placebo 167±132 mg/dl, OE 157±104 mg/dl (p=0.718) | Not measured | Not measured | Not performed |
| Jiang et al., 2021 | ALT & AST: Both significantly elevated in EtOH group vs control (p<0.05). Both RPS and SPS treatments significantly reduced ALT and AST levels vs EtOH group. Strong negative correlations: Lactobacillus reuteri with ALT (r=-0.81, p<0.01) and AST (r=-0.88, p<0.01) | Hepatic TG: Decreased by 17.88% in RPS group and 15.69% in SPS group vs EtOH. Serum TG: Reduced by both treatments but no significant difference between RPS and SPS | GSH, SOD, CAT: All significantly reduced by ethanol, restored by both RPS and SPS treatments. MDA: Significantly increased by ethanol, reduced by polysaccharide treatments | Hepatic TNF-α and IL-1β: Both significantly elevated in EtOH group, significantly reduced by RPS and SPS treatments (p<0.05). Plasma inflammatory markers: LPS, TNF-α, IL-1β all elevated by ethanol and reduced by treatments | Oil Red O staining: Enhanced lipid droplet size and amount in EtOH group, suppressed after RPS and SPS treatment. Hepatic steatosis clearly demonstrated |
| Shi et al., (2015) | In the CCl4-induced acute liver injury model, both AST and ALT levels were significantly elevated in the CCl4-only group (25.21 ± 3.28 U/L and 26.68 ± 3.22 U/L respectively) compared to controls (15.09 ± 3.48 U/L and 17.33 ± 2.57 U/L). Treatment with CGPS-1 at all doses (200, 400, and 800 mg/kg) significantly reduced both AST (20.98 ± 3.49, 19.21 ± 2.59, and 19.17 ± 2.98 U/L respectively, all P < 0.01) and ALT levels (23.40 ± 3.05 at P < 0.05, 20.30 ± 2.70 and 22.10 ± 2.31 U/L at P < 0.01) compared to CCl4-only treatment. In the ethanol-induced chronic liver injury model, ethanol treatment elevated AST (65.14 ± 13.63 U/L) and ALT (29.08 ± 2.57 U/L) compared to controls (32.40 ± 9.38 U/L and 17.92 ± 3.61 U/L). CGPS-1 treatment provided dose-dependent protection, with the highest dose (450 mg/kg) significantly reducing AST to 53.96 ± 7.20 U/L (P < 0.05) and all doses significantly reducing ALT levels (25.19 ± 3.41 U/L at P < 0.05 for 50 mg/kg, 24.26 ± 2.55 U/L and 22.86 ± 3.62 U/L at P < 0.01 for 150 and 450 mg/kg respectively) | Not measured in this study design | Extensive oxidative stress evaluation: Malondialdehyde (MDA) levels as lipid peroxidation marker, Superoxide dismutase (SOD) activity as antioxidant enzyme marker; measured in serum using standardized commercial assay kits | Not specifically measured; focus was on oxidative stress rather than inflammatory cytokines | Comprehensive histopathological examination: Liver tissues fixed in 10% phosphate-buffered neutral formalin, dehydrated in graded alcohol (50-100%), paraffin-embedded, sectioned, H&E stained, examined by light microscopy at 100× magnification |
| Zhang et al., 2014 | ALT (Alanine aminotransferase) and AST (Aspartate aminotransferase) measured by Hitachi 7600-210 automatic biochemistry analyzer. Both significantly elevated in model group, dose-dependently decreased in treatment groups (P<0.05, P<0.01) | TG (Triglyceride) and TCHO (Total cholesterol) measured by automated biochemistry analyzer. Both significantly elevated in model group, progressively decreased from low to high-dose treatment groups | GSH (Glutathione) by DTNB colorimetry: Model 3.45±0.71, Control 6.11±1.35, Low 3.77±0.89, Middle 4.35±1.10, High 5.09±1.39 nmol/g; MDA (Malondialdehyde) by TBA colorimetry: Model 155.20±12.99, Control 105.65±11.25, Low 147.48±19.25, Middle 138.27±16.73, High 98.37±21.09 μmol/g | TNF-α, IL-17, C3a, C5a measured by ELISA. All significantly elevated in model group vs control. Treatment groups showed dose-dependent decreases: TNF-α and IL-17 significantly lower in all treatment groups (P<0.05); C3a and C5a progressively decreased from low to high-dose groups | Liver tissue fixed in 10% formalin, H&E stained 5μm sections. Visual grading system (0-4 points): Model group showed severe lipid accumulation (grade 3-4), middle and high-dose groups demonstrated reduced steatosis with improved hepatocyte architecture |
| Zhao et al., 2019 | AST (Aspartate aminotransferase) and ALT (Alanine aminotransferase) measured using commercial assay kits from Nanjing Jiancheng Bioengineering Institute. Model group: AST 26.057±4.391 IU/L, ALT 13.757±3.137 IU/L; SCGP high-dose: AST 14.789±1.974 IU/L, ALT 9.104±2.229 IU/L (P<0.01) | T-CHO (Total cholesterol): Model 4.450±0.502 mmol/L vs SCGP high-dose 3.866±0.752 mmol/L; LDL-C (Low density lipoprotein cholesterol): Model 4.367±0.427 mmol/L vs SCGP high-dose 3.702±0.535 mmol/L (P<0.05) | Antioxidant activities measured in vitro: DPPH radical scavenging (SCGP 79.7% vs CGP 48.9% at 10 mg/mL), Hydroxyl radical scavenging (SCGP 83.8% vs CGP 45.7% at 4 mg/mL), ABTS radical scavenging (SCGP 81.3% vs CGP 51.3% at 40 mg/mL) | No direct inflammatory cytokine measurements reported - study focused on metabolomic biomarkers and biochemical parameters | Liver tissue H&E staining at ×100 magnification: Control group showed normal hepatocyte arrangement with no pathological changes; Model group developed typical liver injury with inflammation; SCGP treatment groups showed clearly reduced pathological changes from alcoholic injury with dose-dependent improvement |
| Lee et al., 2021 | AST (aspartate aminotransferase), ALT (alanine aminotransferase) | Not measured | GSH (glutathione), MDA (malondialdehyde/LPO - lipid peroxidation), γ-GCS (γ-glutamylcysteine synthetase), GST (glutathione S-transferase), GR (glutathione reductase) | Not measured | Normal: normal hepatic cells with central veins, clear nucleolus, well-distributed cytoplasm; D-GalN: destructive hepatic lobules, necrosis, massive inflammatory infiltration, congestion, extensive vacuolization; Oyster groups: protective effects with reduced pathological changes |
| Wang et al., 2022 | ALT: 83.23→54.82 U/L (↓34.14%, p<0.01); AST: 222.94→144.21 U/L (↓35.31%, p<0.01); ALP: 88.94→73.67 U/L (↓17.18%, p<0.05); TP: 40.63→47.03 g/L (↑17.30%, p<0.05) | Serum TC: ↓21.61% (p<0.01), TG: ↓25.64% (p<0.01), LDL-C: ↓27.91% (p<0.01), HDL-C: ↑33.61% (p<0.05); Hepatic total lipid, TC, TG all significantly decreased | Not directly measured (transcriptome showed regulation of oxidative pathways) | IL-1β, TNF-α, TGF-β all significantly decreased (p<0.01) in liver; LPS decreased in serum (↓12.44%, p<0.05) and liver (↓17.57%, p<0.05) | Liver injury grade ↓31.56% (p<0.01); Oil Red O positive area ↓55.11% (p<0.01); Reduced lipid droplets, fatty vacuolization, inflammatory infiltration |
| Wang et al., 2022 (2) | AST: 19.35→8.47 IU/L (↓56.26%, p<0.01); ALT: 11.64→6.17 IU/L (↓47.04%, p<0.01); GGT: ↓46.34% (p<0.01) | TG: Model group ↑47.90% vs control, OP-M group ↓27.35% vs model (p<0.01) | ROS: ↓36.37% (p<0.01); SOD: ↑40.12% (p<0.01); GSH: ↑44.88% in OP-L (p<0.01); MDA: ↓53.45% (p<0.01) | IL-1β: ↓50.29% in OP-H (p<0.05); IL-6: ↓29.73% in OP-H (p<0.05); TNF-α: ↓46.89% in OP-L (p<0.05) | H&E staining showed reduced lobular inflammation, fatty accumulation, hepatocellular swelling; OP-H group showed well-preserved cytoplasm and legible nucleoli |
| Siregar et al., 2022 | ALT: Vehicle (baseline); EtOH+saline (significantly elevated); EtOH+OBC, EtOH+taurine, EtOH+OH (significantly reduced vs EtOH+saline, p<0.05). AST: Same pattern - elevated in EtOH+saline, significantly decreased in all treatment groups vs EtOH+saline (p<0.05) | Lipid profile not measured in this study | ROS/RNS: EtOH+saline (significantly increased vs vehicle); EtOH+OBC, EtOH+taurine, EtOH+OH (significantly reduced vs EtOH+saline, p<0.05). CYP2E1 activity: High in EtOH+saline; significantly decreased in all treatment groups. Ca2+ concentration: Elevated in EtOH+saline; significantly reduced in treatment groups. Catalase activity: Enhanced in treatment groups | TNF-α, IL-1β, IL-6: Significantly elevated in EtOH+saline vs vehicle; significantly decreased in EtOH+OBC, EtOH+taurine, EtOH+OH vs EtOH+saline (p<0.05). NF-κB: Nuclear translocation observed in EtOH+saline; translocation inhibited in all treatment groups. CD68: Higher macrophage infiltration in EtOH+saline; significantly decreased in treatment groups (p<0.05) | H&E staining: EtOH+saline showed hepatocellular damage with contracted cytoplasm and nucleus, numerous lipid vacuoles in hepatocyte cytoplasm; hepatocellular damage was prevented in EtOH+OBC and EtOH+taurine groups. CD68 immunofluorescence: Higher green fluorescent signals (macrophages) in EtOH+saline; significantly decreased macrophage numbers in treatment groups (n=5, p<0.05) |
| Byun et al., 2021 | ALT: ED group above normal range (>30.2 IU/L); TGPN groups significantly reduced to normal ranges in dose-dependent manner (p<0.05, p<0.01, p<0.001). AST: ED group above normal range (>80.8 IU/L); TGPN groups significantly reduced to normal ranges dose-dependently (p<0.05, p<0.01, p<0.001) | TG: Serum and liver TG significantly elevated in ED vs ND (p<0.01, p<0.001); significantly decreased in TGPN groups vs ED (p<0.05, p<0.01, p<0.001). TC: Serum and liver TC significantly elevated in ED vs ND (p<0.01, p<0.001); significantly reduced in TGPN groups vs ED (p<0.05, p<0.01, p<0.001). HDL/TC ratio: Significantly lower in ED vs ND; significantly increased in TGPN groups vs ED | Oxidative stress markers not directly measured in this study | TNF-α: Serum TNF-α significantly higher in ED vs ND (p<0.001); significantly lower in TGPN groups vs ED (p<0.05, p<0.01, p<0.001). TNF-α mRNA: Liver TNF-α gene expression significantly higher in ED vs ND (p<0.001); significantly decreased in TGPN groups vs ED (p<0.05, p<0.01, p<0.001) | H&E staining: ED group showed significantly higher number of lipid droplets vs ND (p<0.001); TGPN groups showed significantly lower lipid droplets vs ED (p<0.05, p<0.01, p<0.001). Oil Red O staining: Higher lipid accumulation in ED vs ND; TGPN significantly decreased lipid accumulation and restored normal levels (p<0.05, p<0.01, p<0.001) |
| Gao et al., 2022 | ALT: Normal 43.21 → Model 86.12 (p<0.001) → AOPH-H 58.80 (p<0.01); AST: Normal 129.44 → Model 201.5 (p<0.001) → AOPH-H 139.23 (p<0.01); LDH: Model elevated → AOPH-M 395.43 (p<0.01) → AOPH-H 346.63 (p<0.01); ADH: Model decreased → AOPH-H significantly increased (p<0.05) | TC: Model elevated → AOPH-M 3.24 (p<0.01) → AOPH-H 3.26 (p<0.01) (both reduced vs Model) | MDA: Model significantly elevated (p<0.001) → AOPH-H near-normal (p<0.05); T-AOC: Model decreased (p<0.001) → AOPH-H 14.53% increase (p<0.05); GSH-PX: Model decreased → AOPH-H 12.85% increase (p<0.05) | Not measured | H&E and Oil Red O staining showed dose-dependent improvement: hepatocyte swelling reduced, fat droplet accumulation decreased, inflammatory cell infiltration lessened. AOPH-H showed similar/better effects than silymarin |
