# Supplementary material for: Hepatoprotective effects of oyster-derived bioactive compounds in alcoholic liver disease: a systematic review
Source: Front Gastroenterol (Lausanne). 2026 Mar 17;5:1737942. doi: 10.3389/fgstr.2026.1737942 (PMC13035715; doi:10.3389/fgstr.2026.1737942)
Supplement: Supplementary file 1 [file DataSheet1.zip › supplementary/Supplementary Table S4.docx]

SupplementaryTable S4. Histological Changes, Secondary Outcomes, Statistical Analysis, and Clinical Significance of Oyster-Derived Interventions for Alcohol-Related Liver Disease

| **Author(s), year** | **Other Outcomes** | **Significant Improvement?** | **Statistical Methods** | **Key P-values** | **Dose-Response Observed** | **Clinical Significance** |
| --- | --- | --- | --- | --- | --- | --- |
| Osaki et al., 2015 | Safety Profile: OE group had fewer adverse events (7% vs 29%). Common cold: 2 OE vs 4 placebo. Gastrointestinal upset: 0 OE vs 4 placebo. Hangovers: 0 OE vs 2 placebo. Anthropometric: No significant changes in BMI, blood pressure, or heart rate. Compliance: >86.9% in all subjects (average 99.2%). Additional biochemical: Total protein, albumin, total bilirubin, LDH, BUN, creatinine, uric acid, electrolytes, glucose - no significant abnormalities. Taurine content: >5% w/w in oyster extract (>50 mg taurine per daily dose) | Yes - Primary endpoint (GGT) showed significant between-group improvement | Student's unpaired t-test for between-group comparisons, Student's paired t-test for within-group comparisons, χ² test for proportions | Primary outcome: GGT between-group difference at week 12: p=0.049. Within-group GGT changes: Placebo p=0.077 (non-significant increase), OE p=0.290 (non-significant decrease). ALT between-group: Not significant at any timepoint. AST between-group: Not significant at any timepoint | Not evaluated - single dose studied | Results suggest OE supplementation may prevent progression from early alcoholic liver injury (elevated GGT) to more severe ALD stages. Study population appeared to be at early stage of ALD development |
| Jiang et al., 2021 | Gut Microbiota Changes: RPS and SPS significantly increased beneficial bacteria (Lactobacillus reuteri, Bifidobacterium longum, Roseburia spp.) and decreased harmful bacteria (Escherichia, unidentified Clostridiales, Turicibacter). SCFA Production: Both treatments significantly increased propionate and butyrate levels in feces and serum. Gut Barrier Function: Enhanced tight junction proteins (Occludin, ZO-1, Claudin-4) and increased colon length. Molecular Mechanisms: Activated AMPK-α phosphorylation and reduced SREBP-1c expression, indicating improved lipid metabolism. Steaming Effect: No significant difference between RPS and SPS effects, indicating steaming doesn't impair bioactivity. Polysaccharide Characterization: RPS purity 88%, SPS purity 84%. Both contained >50% glycogen. Correlation Analysis: Strong correlations between beneficial bacteria, SCFAs, and improved liver parameters | Yes - Both RPS and SPS showed significant hepatoprotective effects | One-way ANOVA followed by Tukey's post-hoc test for multiple comparisons (SPSS 17.0). 16S rRNA sequencing analyzed separately. Spearman correlation analysis for microbiota-biochemical relationships | Primary outcomes: All liver enzymes, inflammatory markers, and oxidative stress markers showed p<0.05 for treatment vs EtOH groups. Microbiota correlations: Multiple significant correlations (r=-0.74 to -0.92, p<0.01) between beneficial bacteria and liver injury markers | Not evaluated - single dose tested (282 mg/kg). Dose equivalent to practical human consumption (50g oyster meat/day) | Practical Application: Demonstrates that regular oyster consumption (5 oysters/day equivalent) could prevent alcohol-induced liver injury. Cooking Method: Steaming doesn't reduce bioactivity, supporting common culinary practices. Therapeutic Target: Gut microbiota modulation as intervention strategy for ALD. Preventive Medicine: Natural polysaccharide supplementation as alternative to pharmaceutical interventions for early-stage ALD. Mechanistic Insight: First study to demonstrate gut-liver-metabolite axis as primary mechanism for oyster polysaccharide hepatoprotection |
| Shi et al., (2015) | Molecular characterization: UV spectroscopy, FT-IR spectroscopy (peaks at 3352.62, 2931.92, 1632.52, 1079.95, 846.31 cm⁻¹), ¹³C NMR spectroscopy (signals at δ99.73, δ76.75-69.30, δ60.44 ppm), monosaccharide composition analysis | Yes - statistically significant improvements across multiple parameters in dose-dependent manner | Rigorous statistical analysis: One-way analysis of variance (ANOVA) followed by Duncan's multiple-range post-hoc tests using SPSS for Windows Version 13.0; experiments performed in triplicate | Dual significance levels: P<0.05 considered significant, P<0.01 considered very significant; specific results: AST (P<0.05 at 450mg/kg), ALT (P<0.01 at 150 and 450mg/kg), MDA (P<0.01 at 150 and 450mg/kg), SOD (P<0.01 at all doses) | Clear dose-response relationship observed: 450mg/kg > 150mg/kg > 50mg/kg for most parameters; optimal hepatoprotective effects at highest tested dose | High clinical relevance: CGPS-1 demonstrated potent hepatoprotective activity against both acute and chronic liver injury models, suggesting potential therapeutic application for alcohol-related liver disease; novel extraction method offers economic advantages for industrial production |
| Zhang et al., 2014 | Plasma Zn levels: Model group significantly decreased vs control (P<0.01), dose-dependent increases in treatment groups (P<0.05); Anti-MAA-HSA IgG: Elevated in model group, significantly decreased in middle/high-dose groups (P<0.01). T-cell subsets and NK cell analysis by flow cytometry: CD3+ (Model 45.7±7.0% vs High-dose 57.0±7.1%, P<0.05), CD4+ (Model 33.1±4.8% vs High-dose 41.5±7.8%, P<0.05), CD8+ decreased in high-dose (33.8±3.8% vs Model 38.4±6.9%, P<0.01), NK cells increased in treatment groups | Yes - statistically significant improvements across multiple parameters including hepatic enzymes, lipid metabolism, oxidative stress, inflammatory markers, immune function, and histological architecture | SPSS 11.5 statistical software, One-Way ANOVA for measurement data analysis, Mann-Whitney Test for pathological changes comparison between groups, all data expressed as mean ± SD | Primary comparisons: P<0.05 for significant differences, P<0.01 for highly significant differences. Most treatment group improvements vs model group achieved P<0.01 significance level | Clear dose-response relationship observed across all measured parameters: low-dose showed modest improvements, middle-dose demonstrated significant benefits, high-dose achieved optimal therapeutic effects with strongest statistical significance | This study provides compelling preclinical evidence for oyster extract as a multi-mechanistic hepatoprotective agent against alcoholic liver disease. The extract demonstrates: (1) Hepatocellular protection via enzyme normalization, (2) Metabolic restoration through lipid profile improvement, (3) Antioxidant activity via GSH enhancement and MDA reduction, (4) Anti-inflammatory effects through cytokine modulation, (5) Immunomodulatory benefits via T-cell and NK cell optimization, (6) Nutritional supplementation through zinc replenishment, (7) Structural liver protection evidenced by histological improvement. The dose-dependent efficacy, safety profile, and multiple therapeutic targets suggest strong translational potential for clinical ALD management, particularly given oyster extract's status on the medicine-food homology list and established safety record. |
| Zhao et al., 2019 | TBIL (Total bilirubin): Model 4.209±0.879 μmol/L vs SCGP high-dose 2.545±0.411 μmol/L (P<0.01); Acute toxicity test showed LD₅₀ >10 g/kg body weight with no toxic effects observed over 14 days. Metabolomics Analysis: 21 potential biomarkers identified by UHPLC-Q-TOF-MS including L-Pyroglutamic acid, adenosine, adenine, cytosine, corticosterone, L-Glutamine, acetylcarnitine, amino acids (L-Leucine, L-Arginine, L-Isoleucine, L-Tyrosine, L-Valine), allantoin, L-Ascorbic acid. Structural Characterization: FT-IR confirmed sulfation (peaks at 1275 cm⁻¹ and 811 cm⁻¹), ¹³C NMR showed sulfate substitution primarily at C-6 position | Yes - statistically significant improvements in all measured biochemical parameters (T-CHO, LDL-C, TBIL, AST, ALT) and antioxidant activities compared to model group. Metabolomic analysis revealed significant metabolic pathway alterations | EXCEL (Microsoft) and SPSS 20.0 for statistical analysis. Data presented as mean ± SD from triplicate experiments. OPLS-DA analysis for metabolomics with R² = 0.883-0.919 and Q² = 0.393-0.600 indicating reliable model | P<0.05 considered significant, P<0.01 highly significant. Most treatment comparisons vs model group achieved P<0.01 significance. Metabolomic biomarkers identified based on VIP>1 and P<0.05 | Clear dose-response relationship: Low dose (100 mg/kg) showed modest improvements, medium dose (200 mg/kg) demonstrated significant benefits, high dose (400 mg/kg) achieved optimal therapeutic effects across all measured parameters | This study provides comprehensive evidence for sulfated oyster polysaccharides as a novel hepatoprotective agent with superior bioactivity compared to native polysaccharides. Key clinical implications include: (1) Enhanced Bioactivity: Sulfation modification significantly improved antioxidant activities (DPPH: 79.7% vs 48.9%, hydroxyl radical: 83.8% vs 45.7%, ABTS: 81.3% vs 51.3%) compared to native polysaccharides. (2) Multi-target Therapeutic Effects: SCGP demonstrated hepatoprotection through multiple mechanisms including liver enzyme normalization, lipid metabolism improvement, and metabolic pathway restoration. (3) Metabolomic Insights: First study to use metabolomics to identify 21 biomarkers associated with amino acid metabolism, oxidative stress, and immune response, providing mechanistic understanding of hepatoprotective effects. (4) Safety Profile: Excellent safety with LD₅₀ >10 g/kg and no toxic effects, supporting clinical translation potential. (5) Structure-Activity Relationship: Confirmed sulfate substitution primarily at C-6 position with optimal DS of 0.66, providing guidance for pharmaceutical development. The study establishes SCGP as a promising therapeutic candidate for alcoholic liver disease with strong mechanistic rationale and safety profile. |
| Lee et al., 2021 | ADH (alcohol dehydrogenase) and ALDH (acetaldehyde dehydrogenase) activities in liver, blood ethanol concentration measured at 30, 60, 120, 180, 300 minutes, phenolic compounds (gallic acid, chlorogenic acid, gentisic acid), amino acid profiles, monosaccharide content | Yes - SOP showed superior hepatoprotective effects compared to OP in both D-GalN-induced liver injury and alcohol metabolism studies | Student's t-test using SPSS Statistics 17.0; all values expressed as mean ± SD | P < 0.05 considered statistically significant (specific p-values not reported, only indicated by * in figures) | Yes - optimal dose 250 mg/kg determined from dose-response study (100, 250, 500 mg/kg tested); 250 mg/kg showed best balance of efficacy and safety based on body weight, AST, ALT results | SOP demonstrated potential as functional food for preventing alcohol hangover and treating D-GalN-induced hepatitis; subcritical water processing enhanced bioactive compounds and hepatoprotective efficacy |
| Wang et al., 2022 | Liver weight decreased (p<0.01), body weight increased, transcriptome/proteome analysis revealed 43 co-regulated targets | Yes, dose-dependent improvements across all parameters | One-way ANOVA, Tukey's post hoc test, p<0.05 significant | Most significant results at p<0.01 level for high-dose group; medium dose showed p<0.05 for some markers | Clear dose-response: Low (200mg/kg) < Medium (400mg/kg) < High (800mg/kg) effects | High dose effects comparable to silymarin positive control, suggesting clinical potential. 482 target genes and 111 target proteins involved; reduced hepatic steatosis and inflammation |
| Wang et al., 2022 (2) | Liver index decreased by 13.67%, 9.97%, 13.20% in OP-L, OP-M, OP-H respectively (p<0.05); mRNA expression changes in Nrf2, HO-1, NQO1 pathways | Yes, dose-dependent improvements across all parameters | One-way ANOVA, Duncan's multiple comparison, p<0.05 significant | Most liver enzymes p<0.01, oxidative markers p<0.01, inflammatory markers p<0.05 | Clear dose-response: Low (120mg/kg) < Medium (240mg/kg) < High (480mg/kg) | High dose showed substantial improvements comparable to positive control (dimethyl diphenyl bicarboxylate). OP significantly improved liver enzymes, reduced oxidative stress, decreased inflammation, and restored liver histology with dose-dependent effects |
| Siregar et al., 2022 | Alcohol metabolism: Blood EtOH concentration significantly decreased, ADH/ALDH/catalase activities enhanced in treatment groups. Apoptosis: Bax/Bcl-2 ratio, cytochrome C release, caspase-3 cleavage reduced in treatment groups. ER stress: GRP78, p-PERK, p-eIF2α, ATF4, ATF6, CHOP upregulated in EtOH+saline, downregulated in treatment groups. Motor function: Rotarod performance significantly improved in treatment groups vs EtOH+saline | YES - OBC and taurine showed comparable hepatoprotective effects to OH (positive control). OBC effects attributed to high taurine content (41.8% of total free amino acids). Taurine alone showed no effects under normal conditions (vehicle vs taurine alone: no significant differences) | One-way ANOVA with Bonferroni post-hoc test or Kruskal-Wallis with Mann-Whitney U test (selected after normality testing using OriginPro2020). Sample sizes: n=15 for behavioral tests, n=12 for enzyme assays, n=5 for histology, n=6 for cytokine analysis, n=3 for western blots | p < 0.05 considered statistically significant for all comparisons. All treatment groups vs EtOH+saline group showed p<0.05 for: liver enzymes (ALT, AST), oxidative stress markers (ROS/RNS, CYP2E1, Ca2+), inflammatory markers (TNF-α, IL-1β, IL-6, CD68), and histological improvements | No dose-response relationship tested - single doses used: OBC (200 mg/kg), taurine (45 mg/kg), OH (200 mg/kg, positive control). Taurine concentration in OBC was 37.54 mg/kg, similar to pure taurine dose | High clinical significance - OBC is economically valuable (by-product of oyster canning industry), simple production method (3-min boiling at 95°C), rich in taurine and other beneficial amino acids. Strong potential as functional food for alcohol-induced liver damage prevention. Multiple protective mechanisms: enhanced alcohol metabolism, reduced ER stress, anti-inflammatory and anti-apoptotic pathways. OBC showed hepatoprotective effects less than OH but considering economics, OBC is as valuable as OH |
| Byun et al., 2021 | Liver index: ED group significantly higher vs ND; significantly decreased in TGPN and silymarin groups vs ED (p<0.05). Adiponectin: Serum level significantly lower in ED vs ND; significantly increased in TGPN groups vs ED. Body weight: ED group showed lower body weight gain; TGPN increased gain but not significantly | YES - TGPN showed significant improvement in all measured parameters. Effects comparable to silymarin (positive control). TGPN promoted AMPK and PPAR-α activation, inactivated ACC, decreased FAS and SCD1, increased CPT-1, and decreased SREBP-1c and SREBP-2 expression. Mechanism involved inhibition of fatty acid synthesis, promotion of fatty acid oxidation, and inhibition of TG and cholesterol synthesis | Shapiro-Wilk normality test followed by one-way ANOVA with Tukey's post hoc test using SPSS version 25. Sample size n=10 per group for body weight measurements, n=6 per group for biochemical and histological analyses | Significance levels: ###P<0.001, ##P<0.01 vs ND group; ***P<0.001, **P<0.01, *P<0.05 vs ED group. All treatment groups showed significant improvements vs ED group in liver enzymes (ALT, AST), lipid profile (TG, TC), inflammatory markers (TNF-α), and histological parameters | YES - Clear dose-response relationship observed: TGPN 50 < TGPN 100 < TGPN 200 for most parameters including ALT, AST reduction and lipid profile improvement. Higher doses showed greater statistical significance (p<0.001 vs p<0.05 for lower doses) | High clinical significance - TGPN ameliorated alcoholic fatty liver through multiple mechanisms: enhanced AMPK/PPAR-α pathway activation, inhibited ACC activity, decreased fatty acid and cholesterol synthesis, promoted fatty acid oxidation, reduced liver damage markers, and decreased inflammation. The study identified specific molecular targets (AMPK, ACC, SREBP-1c/2) and downstream effects on lipid metabolism genes (FAS, SCD1, CPT1, HMGR, LDLR). TGPN represents a potential therapeutic intervention for alcoholic fatty liver disease with established mechanism of action |
| Gao et al., 2022 | Liver index: Model 4.62% vs Normal 4.13%, AOPH-H reduced to 4.19%. Molecular analysis: 834 DEGs, 54 DAPs in oxidative phosphorylation, glutathione metabolism, PPAR signaling, cytochrome P450 pathways | Yes, particularly at high dose (800mg/kg) with dose-dependent effects across all parameters | One-way ANOVA with Duncan's multiple comparison test | p<0.05, p<0.01, p<0.001 | Yes, clear dose-response relationship observed across liver enzymes, oxidative markers, and histological improvements | High dose (800mg/kg) most effective, providing hepatoprotection comparable to positive control silymarin |
