# Supplementary material for: Hepatoprotective effects of oyster-derived bioactive compounds in alcoholic liver disease: a systematic review
Source: Front Gastroenterol (Lausanne). 2026 Mar 17;5:1737942. doi: 10.3389/fgstr.2026.1737942 (PMC13035715; doi:10.3389/fgstr.2026.1737942)
Supplement: Supplementary file 1 [file DataSheet1.zip › supplementary/Supplementary table S5.docx]

**Supplementary Table S5:** Risk of bias assessment for included studies. Domain-level judgments are presented for animal studies (assessed with the SYRCLE Risk of Bias tool) and the single randomized controlled trial (assessed with the Cochrane Risk of Bias 2.0 tool). Ratings are categorized as *low risk*, *high risk*, or *unclear risk*.

| **Study** | **Tool Used** | **Seq. Gen.** | **Baseline** | **Alloc. Conceal.** | **Random Housing** | **Caregiver Blinding** | **Outcome Assessment (Random/Blind)** | **Incomplete Data** | **Selective Reporting** | **Other Bias** | **Overall RoB** |
| --- | --- | --- | --- | --- | --- | --- | --- | --- | --- | --- | --- |
| Jiang et al. 2021 | SYRCLE | Unclear | Low | Unclear | Unclear | High | Unclear / High | Low | Low | Low | High |
| Shi et al. 2015 | SYRCLE | Unclear | Low | Unclear | Low | Unclear | Unclear / Unclear | Low | Low | Low | Unclear |
| Zhang et al. 2014 | SYRCLE | Unclear | Low | Unclear | Unclear | High | Low / High | Low | Low | Unclear | High |
| Zhao et al. 2019 | SYRCLE | Unclear | Low | Low | Unclear | High | Unclear / High | Low | Low | Low | High |
| Lee et al. 2021 | SYRCLE | Unclear | Low | Unclear | Unclear | High | Unclear / High | Low | Low | Low | High |
| Wang et al. 2022 (a) | SYRCLE | Low | Low | Unclear | Low | High | Unclear / Unclear | Low | Low | Low | Unclear |
| Wang et al. 2022 (b) | SYRCLE | Unclear | Low | Unclear | Unclear | High | Low / High | Low | Low | Low | High |
| Siregar et al. 2022 | SYRCLE | Unclear | Low | Low | Unclear | High | Unclear / High | Low | Low | Low | High |
| Byun et al. 2021 | SYRCLE | Unclear | Low | Unclear | Unclear | Unclear | Unclear / Unclear | Low | Low | Low | Unclear |
| Gao et al. 2022 | SYRCLE | Unclear | Low | Unclear | Unclear | Unclear | Unclear / Unclear | Low | Low | Low | Unclear |
| Osaki et al. 2015 | Cochrane RoB 2.0 | Low | – | – | – | – | Low / Low | Low | Low | – | Low |
